# Influence of omega-3 fatty acids on skeletal muscle protein metabolism and mitochondrial bioenergetics in older adults

**DOI:** 10.18632/aging.101210

**Published:** 2017-04-05

**Authors:** Antigoni Z. Lalia, Surendra Dasari, Matthew M. Robinson, Hinnah Abid, Dawn M. Morse, Katherine A. Klaus, Ian R. Lanza

**Affiliations:** ^1^ Division of Endocrinology and Metabolism, Mayo Clinic College of Medicine, Rochester, Minnesota, USA

**Keywords:** n3-PUFA, EPA, DHA, sarcopenia, protein synthesis, mitochondria

## Abstract

Omega-3 polyunsaturated fatty acids (n3-PUFA) are recognized for their anti-inflammatory effects and may be beneficial in the context of sarcopenia. We determined the influence of n3-PUFA on muscle mitochondrial physiology and protein metabolism in older adults. Twelve young (18-35 years) and older (65-85 years) men and women were studied at baseline. Older adults were studied again following n3-PUFA supplementation (3.9g/day, 16 weeks). Muscle biopsies were used to evaluate respiratory capacity (high resolution respirometry) and oxidant emissions (spectrofluorometry) in isolated mitochondria. Maximal respiration was significantly lower in older compared to young. n3-PUFA did not change respiration, but significantly reduced oxidant emissions. Participants performed a single bout of resistance exercise, followed by biopsies at 15 and 18 hours post exercise. Several genes involved in muscle protein turnover were significantly altered in older adults at baseline and following exercise, yet muscle protein synthesis was similar between age groups under both conditions. Following n3-PUFA supplementation, mixed muscle, mitochondrial, and sarcoplasmic protein synthesis rates were increased in older adults before exercise. n3-PUFA increased post-exercise mitochondrial and myofibrillar protein synthesis in older adults. These results demonstrate that n3-PUFA reduce mitochondrial oxidant emissions, increase postabsorptive muscle protein synthesis, and enhance anabolic responses to exercise in older adults.

## INTRODUCTION

Skeletal muscle is critical to physical function, health, and quality of life throughout the lifespan, but muscle mass and strength are clearly compromised in older adults [1], especially after the 6^th^ decade of life [2]. Sarcopenia is a major factor contributing to decreased physical function in older adults and predictive of future disability [3]. Sarcopenia has multifactorial origins, and its negative influence on healthspan is compounded by other related hallmarks of aging, which include reduced whole-body peak oxygen uptake (VO_2_ peak) [4], neurodegenerative changes, increased adiposity [5, 6], decreased regenerative potential of the muscle stem cell niche [7, 8], and changes in mitochondrial physiology [9-12].

Exercise is a proven therapeutic strategy to prevent and reverse sarcopenia and related complications [10, 13], with pleiotropic adaptations that include improvements in muscle mass and strength [14], increased muscle protein synthesis rates [15, 16], mitochondrial proliferation [17, 18], increased oxidative capacity [10], and increased satellite cell content [19] in older adults. However, in many cases these adaptations are blunted, slower to occur, and more rapidly lost after exercise in older adults [15, 18, 20, 21]. For example, older adults exhibit blunted hypertrophic responses to resistance exercise training [22, 23] and attenuated increases in muscle protein synthesis in response to acute bouts of resistance exercise [24-26]. These observations are consistent with the concept of anabolic resistance with aging [27], which appears to be driven, in part, by chronic, low grade, sterile inflammation that is often observed with aging [28, 29] and linked with frailty [30]. It is known that inflammatory cytokines such as tumor necrosis factor ɑ (TNFɑ) inhibit muscle protein synthesis by decreasing mRNA translation efficiency [31]. Furthermore TNFɑ expression was inversely related to muscle protein synthesis in frail elderly [32]. Similarly, Toth et al. showed that postabsorptive muscle protein synthesis rates were 20% lower in older compared to young adults, and inversely related to circulating C-reactive protein, interleukin-6, and TNFɑ receptor II [33]. Together, these studies suggest that inflammation may attenuate anabolic responsiveness in older adults. Further support for this hypothesis comes from studies where inflammation was reduced using non-steroidal anti-inflammatory drugs or omega-3 polyunsaturated fatty acids. These interventions restored protein synthesis rates with aging [34, 35] and enhanced muscle mass and strength gains in response to resistance exercise in older humans [36-38].

There is also solid evidence linking chronic inflammation with mitochondrial abnormalities observed in aging skeletal muscle such as increased oxidative stress [39], and mitochondrial dysfunction due to reduced mitochondrial oxidative capacity [12], abundance [12] and mitochondrial protein expression [10]. Inflammatory mediators such as TNFɑ and IL-1β have been shown to induce reactive oxygen species (ROS) production and alter the activity of mitochondrial respiratory chain complexes [40]. In light of recent reports that dietary omega-3 polyunsaturated fatty acids (n3-PUFA) reduce adipose tissue inflammation in humans [41] and repress TNFɑ signaling pathways in mice [42], we recently determined if dietary n3-PUFA could restore muscle mitochondrial function in aging mice [43]. We reported that the anti-inflammatory effects of eicosapentaenoic acid were associated with upregulated transcriptional regulators of mitochondrial biogenesis [44] and marked improvements in mitochondrial function in old mice [43]. It remains unclear whether similar nutritional strategies to suppress chronic inflammation in older humans will improve anabolic responsiveness to exercise or recapitulate the improvements in muscle mitochondrial function observed in rodents.

**Figure 1:**
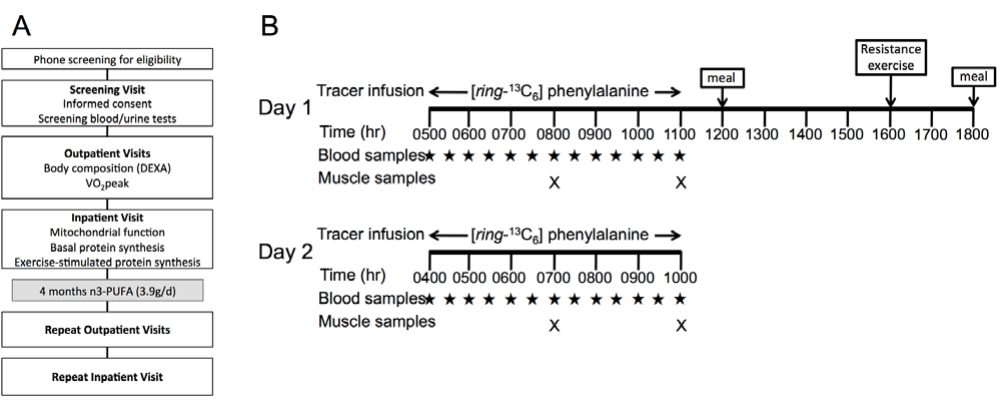
Study design Study design (**A**) involved an initial phone screening, screening visit, outpatient testing for body composition and physical function, and inpatient testing of mitochondrial physiology and muscle protein metabolism. Older adults repeated study visits following 16 weeks of n3-PUFA supplementation. Inpatient study procedures (**B**) involved basal postabsorptive muscle protein synthesis measurements on Day 1 by infusing ^13^C_6_-Phenylalanine and serial muscle biopsies on the right leg. Following a standardized meal, a single bout of unaccustomed resistance exercise was performed at 1600 hr using the left leg. A second tracer infusion began on day 2, followed by serial biopsies to assess muscle protein synthesis 15-18 hours following exercise.

In the current study, we sought to translate early observations from mice to human aging by determining the influence of dietary n3-PUFA on muscle mitochondrial physiology, skeletal muscle protein synthesis, and anabolic response to acute exercise in healthy older adults. Twelve healthy young (18-35 years) and older (65-85 years) men and women were studied at baseline. Older adults were studied again following a 16-week open-label intervention of n3-PUFA supplementation at 3.9g/day. Skeletal muscle biopsies were acquired to evaluate the primary outcomes, which included mitochondrial respiration and ROS production, postabsorptive muscle protein synthesis rates, and the anabolic response to an acute bout of exercise (Figure 1).

## RESULTS

The descriptive characteristics of the young and older adults (pre and post n3-PUFA supplementation) are shown in Table 1; the biochemical and metabolic parameters in Table 2, and the plasma free fatty acids profile in Table 3.

**Table 1:** Descriptive characteristics

	Young N=12	Old N=12	Old n-3 N=12	*P_Age_*	*P_Intervention_*
**Physical characteristics**					
Age (years)	27 ± 5	76 ± 5	76 ± 5		
Sex (F/M)	6F/6M	7F/5M	7F/5M		
Height (cm)	169.6 ± 3.7	163.7 ± 8.5	163.7 ± 8.5	0.03	
Weight (kg)	69.9 ± 7.0	70.7 ± 12	71.8 ± 12	0.08	0.02
BMI (kg/m^2^)	24.3 ± 2.7	26.3 ± 2.8	26.6 ± 3.0	0.10	0.02
SBP (mmHg)	112.4 ± 11.8	124.2 ± 11.6	118.4 ± 12.1	0.02	0.05
DBP (mmHg)	69.9 ± 9.7	64.3 ± 10.5	68.8 ± 12.8	0.18	0.26
**Body composition**					
Total body Fat (%)	31.7 ± 7.7	36.6 ± 5.9	36.9 ± 6.2	0.10	0.44
Fat, arms (kg)	2.0 ± 0.5	2.5 ± 0.7	2.7 ± 0.9	0.04	0.004
Fat, legs (kg)	7.6 ± 2.2	7.8 ± 2.2	8.1 ± 2.4	0.82	0.02
Fat, trunk (kg)	10.6 ± 2.7	13.7 ± 5.1	13.9 ± 5.3	0.08	0.41
Total lean mass (kg)	45.5 ± 7.8	42.5 ± 7.3	43.1 ± 7.1	0.30	0.04
Lean, arms (kg)	5.0 ± 1.2	4.5 ± 1.1	4.4 ± 1.1	0.31	0.27
Lean, legs (kg)	15.6 ± 3.1	13.9 ± 2.5	14.1 ± 2.4	0.15	0.20
Lean, trunk (kg)	22 ± 3.6	21.1 ± 3.6	21.5 ± 3.4	0.51	0.05
**Physical function**					
SMI (kg/m^2^)	15.8 ± 2.6	15.8 ± 1.4	16.0 ± 1.3	0.67	0.03
Work (Watts)	155.1 ± 28.4	111.5 ± 37.3	109.3 ± 38.3	0.02	0.80
VO_2_ peak (L/min)	2.2 ± 0.5	1.5 ± 0.4	1.5 ± 0.5	0.006	0.10
VO_2_ peak (mL/kg/min)	31.4 ± 5.6	21.7 ± 4.2	21.4 ± 4.8	<0.001	0.55

M; Male, F; Female, BMI; body mass index, SBP; systolic blood pressure, DBP; diastolic blood pressure, SMI; total skeletal muscle index, 1RM; 1 repetition maximum, AU; arbitrary units, VO_2_ peak; peak oxygen uptake. Data are shown as mean ± SD or median (interquartile range), * p ≤0.05, Wilcoxon rank sum test for age group comparisons and for non-parametric distributions, paired t-test for the intervention.

**Table 2:** Biochemical and metabolic parameters

	Young N=12	Old N=12	Old n-3 N=12	*P_Age_*	*P_Intervention_*
**Glucose and insulin**					
Glucose (mg/dL)	84.3 ± 7.2	91.6 ± 8.6	96.8 ± 8.3	0.04	0.02
Insulin (μIU/mL)	8.3 ± 5.6	7.3 ± 4.2	4.2 ± 1.9	0.60	0.01
HOMA-IR	1.7 ± 1.1	1.7 ± 1.0	1.1 ± 0.6	0.98	0.01
**Lipid panel**					
Cholesterol (mg/dL)	165.1 ± 26.1	194.9 ± 32.1	176.3 ± 28.4	0.02	0.001
LDL (mg/dL)	90.6 ± 21.6	108.1 ± 25.5	105.3 ± 24.3	0.08	0.46
HDL (mg/dL)	58.0 ± 13.2	67.6 ± 12.1	55.5 ± 10.1	0.08	<0.001
TG (mg/dL)	82.3 ± 44.5	95.83 ± 40.3	76.9 ± 19.7	0.40	0.03
**Inflammatory markers**				
IL-6 (pg/mL)	3.0 (2.6, 4.0)	6.15 (4.5, 14.5)	7.6 (6.4, 9.4)	0.03	0.25
CRP (mg/dL)	0.11 (0.06, 0.19)	0.07 (0.05, 0.27)	0.11 (0.07, 0.28)	0.86	0.79
TNF-a (pg/mL)	0.5 (0.5, 0.5)	0.74 (0.5, 0.9)	0.70 (0.5, 1.0)	0.03	1.00
Leptin (ng/mL)	7.7 (3.6, 17.7)	13.9 (4.1, 18.1)	10.6 (4.4, 17.8)	0.54	0.87
Adiponectin (ng/mL)	2836 (1739, 3910)	5629 (4620, 11788)	5396 (4098, 7931)	0.01	0.06
**Energy expenditure**	N=12	N=11	N=11		
RQ	0.83 ± 0.07	0.80 ± 0.03	0.82 ± 0.05	0.32	0.39
REE	1447.3 ± 230.7	1263.5 ± 236.9	1279.8 ± 238.6	0.09	0.80

LDL; low density lipoprotein-cholesterol, HDL; high density lipoprotein-cholesterol, TG; triglycerides, IL-1β; interleukin 1 beta, IL-6; interleukin 6, CRP; c-reactive protein, TNF-α; tumor necrosis factor alpha, RQ; Respiratory quotient, REE; resting energy expenditure. Data are shown as mean ± SD or median (interquartile range), * p ≤0.05, Wilcoxon rank sum test for age group comparisons and for non-parametric distributions, paired t-test for the intervention.

**Table 3:** Plasma Free Fatty Acids profile

	Young N=12	Old N=11	Old n-3 N=11	*P_age_*	*P_Intervention_*
**n3-PUFA**	17.0 ± 9.0	24.1 ± 10.9	59.0 ± 18.9	0.16	<0.0001
EPA	2.8 ± 1.5	3.7 ± 2.2	34.3 ± 12.0	0.32	<0.0001
DHA	3.9 ± 2.5	4.3 ± 1.6	10.1 ± 2.2	0.24	<0.0001
ALA	10.3 ± 6.2	16.1 ± 7.7	14.6 ± 6.1	0.09	0.04
**n6-PUFA**	82.3 ± 42.1	135.5 ± 54.3	117.1 ± 38.8	0.02	0.02
Arachidonic	4.5 ± 1.1	4.3 ± 1.4	4.3 ± 1.1	0.52	0.90
Linoleic	77.8 ± 41.6	131.2 ± 53.0	112.8 ± 38.2	0.02	0.02
**MUFA**	188.4 ± 101.4	274.4 ± 91.0	240.1 ± 66.9	0.02	0.08
Palmitoleic	15.3 ± 11.7	22.8 ± 13.0	20.9 ± 10.9	0.03	0.28
Oleic	173.1 ± 90.4	251.6 ± 79.7	219.3 ± 57.3	0.02	0.07
**SFA**	167.0 ± 68.9	219.7 ± 90.0	193.9 ± 55.9	0.19	0.12
Myristic	8.8 ± 4.4	11.2 ± 5.2	9.9 ± 3.9	0.20	0.03
Palmitic	115.8 ± 55.1	159.5 ± 69.3	139.1 ± 45.3	0.15	0.09
Stearic	42.5 ± 12.5	49.0 ± 16.3	45.0 ± 9.2	0.36	0.34
**Trans Fatty acids**					
Elaidic	5.7 ± 3.0	7.9 ± 3.9	6.8 ± 2.2	0.20	0.13
**n6- : n3- PUFA Ratio**	5.0 ± 1.4	6.1 ± 1.6	2.0 ± 0.3	0.10	<0.0001
**RBC n3-PUFA content**					
EPA (μg/mL)	ND	17.3 ± 7.1	68.6 ±6.4		<0.0001
DHA (μg/mL)	ND	78.0 ± 6.5	127.6 ± 8.9		<0.0001

Following 4 months of n3-PUFA supplementation, n-3 PUFA significantly increased, while decreasing n-6 PUFA, and specifically linoleic acid. PUFA; polyunsaturated fatty acids, ALA; alpha linolenic acid, MUFA; monounsaturated fatty acids, SFA; saturated fatty acids * P ≤ 0.05, Wilcoxon rank sum test for age group comparisons, paired t-test for intervention. Data are presented as mean ± SD.

### Evidence of impaired mitochondrial function in older adults

**Figure 2:**
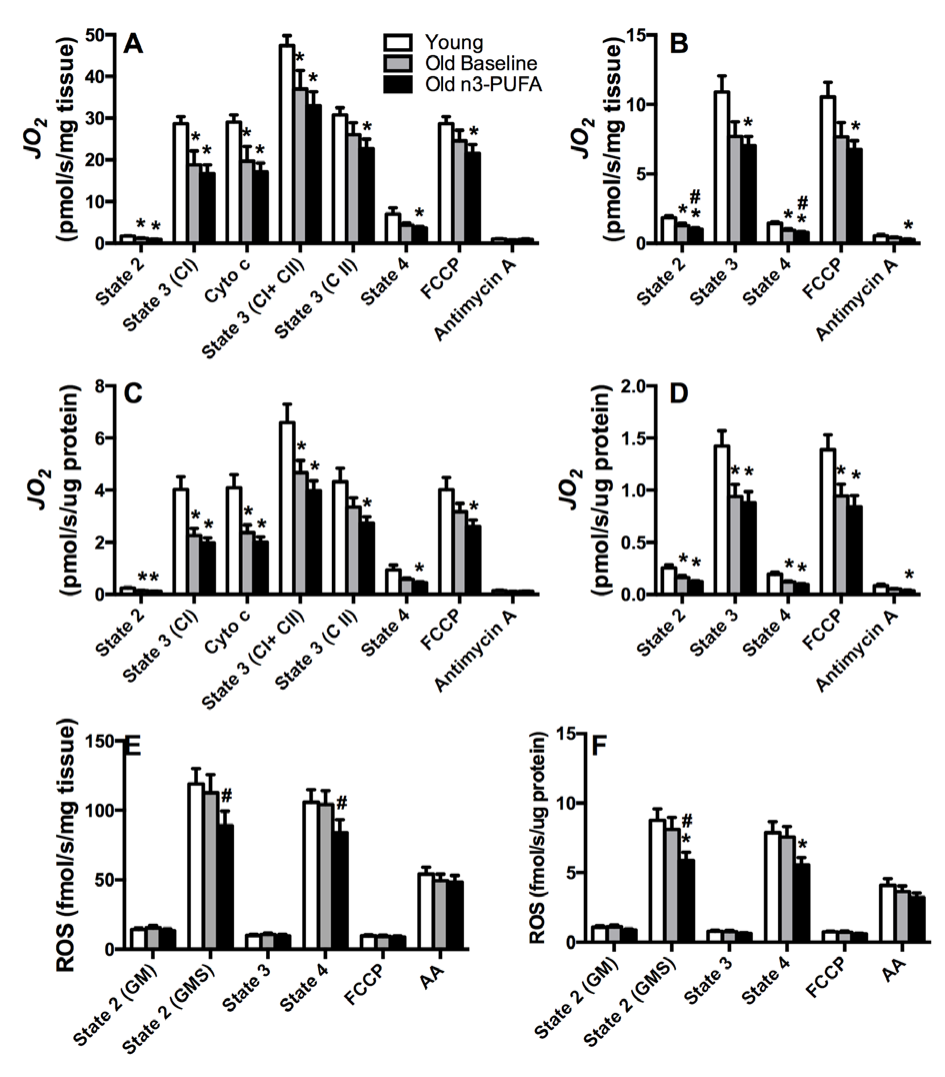
Mitochondrial physiology Respiration of isolated mitochondria was measured by high-resolution respirometry using substrates providing electron flow from carbohydrate based substrates (glutamate, malate, succinate) (**A, C**) and lipid substrates (palmitoyl carnitine, malate) (**B, D**). Older adults exhibited lower skeletal muscle mitochondrial oxidative capacity compared to young adults, with no effect of n3-PUFA supplementation regardless of whether respiration was expressed per tissue wet weight (**A, B**) or mitochondrial protein content (**C, D**). Mitochondrial reactive oxygen species (ROS) production was measured by spectrofluorometric monitoring of Amplex Red oxidation (**E, F**). ROS production was similar in young and old at baseline, but n3-PUFA significantly decreased ROS production in older adults when expressed per tissue wet weight (**E**) and normalized to mitochondrial protein content (**F**). *JO_2_*; mitochondrial oxygen consumption. Cyto c; cytochrome c, control of mitochondrial membrane integrity, FCCP; carbonyl cyanide-p-trifluoro-methoxy-phenyl-hydrazone, a chemical uncoupler, AA; Antimycin A, complex III inhibitor. GM; glutamate+malate. GMS; glutamate+malate+succinate. * Significantly (p≤0.05) different from young. # Significantly different from old baseline. Data bars are mean ± SEM.

Mitochondria were isolated from *vastus lateralis* muscle biopsies under postabsorptive conditions, and oxygen consumption was measured during a stepwise titration protocol using substrates supporting carbohydrate-based respiration (Figure 2A, C) or lipid-based respiration (Figure 2B, D). These experiments revealed that oxygen consumption rates were significantly lower in muscle from older compared to young adults when expressed relative to tissue weight (Figure 2A, B) or relative to mitochondrial protein abundance (Figure 2C, D). This 30-40% reduction in state 3 respiration was primarily evident when respiration was supported through complex I electron flow whereas there was no significant difference in state 3 respiration under conditions where respiration was supported exclusively through complex II. Importantly, the age-related reductions in state 3 respiration persisted when oxygen flux was normalized to mitochondrial protein content (Figure 2C, D), indicating that an age-related reduction in mitochondrial abundance cannot completely explain the loss of mitochondrial oxidative capacity in older adults. The data point to an intrinsic loss in mitochondrial capacity at the organelle level. Despite significant reductions in mitochondrial respiratory capacity, young and older adults exhibited similar rates of hydrogen peroxide (H_2_O_2_) emissions from isolated mitochondria, measured by fluorescence-based monitoring of amplex red oxidation under different energetic states (Figure 2E, F). Regardless of whether H_2_O_2_ was expressed relative to tissue weight (Figure 2E) or mitochondrial protein content (Figure 2F), there was no evidence of an increase in ROS production in mitochondria from older adults.

### n3-PUFA do not increase mitochondrial respiration, but reduce mitochondrial ROS production in older adults

Sixteen weeks of high dose (3.9g/day) n3-PUFA in older adults did not significantly change muscle mitochondrial respiration rates under any of the observed experimental conditions with carbohydrate-based substrates (Figure 2A, C). Although state 2 and state 4 respiration with lipid substrates were significantly lower following n3-PUFA (Figure 2B), these differences did not persist when normalized to mitochondrial protein content (Figure 2D), nor were there any changes in state 3 or fully uncoupled respiration following the intervention. After 4 months of n3-PUFA consumption, there was a 20-25% reduction in mitochondrial H_2_O_2_ production under conditions where the ROS-emitting potential of mitochondria was highest (state 2, state 4). This reduction in ROS production was evident when rates were expressed relative to tissue weight (Figure 2E) or relative to mitochondrial protein content (Figure 2F). The observation that normalizing to mitochondrial protein did not abolish the reduction in ROS production suggests that n3-PUFA reduce intrinsic mitochondrial ROS generation.

### Transcriptional evidence of blunted exercise responsiveness in older adults

**Figure 3:**
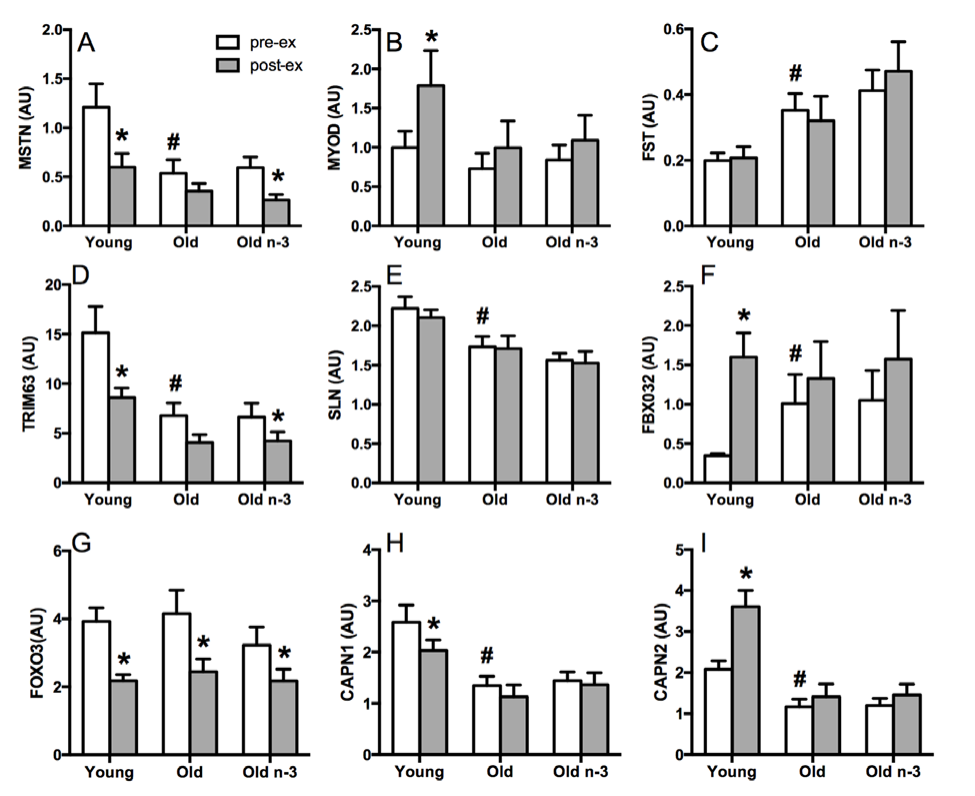
Selected gene transcripts related to muscle protein turnover Gene expression was measured in skeletal muscle by RT-PCR. At baseline, pre-exercise, older adults demonstrated lower expression of myostatin (**A**), tripartite motif containing 63 (**D**), sarcolipin (**E**), and calpain 1 (**H**). Calpain 2 (**I**), follistatin (**C**) and F-box protein 32 (**F**) were higher in older adults at baseline. Several genes exhibited significant changes in expression with exercise in young but not old (**A, B, D, F, H, I**) with no effects of n3-PUFA. FOXO3B; forkhead box O3B pseudogene, forkhead box O3, MSTN; myostatin, TRIM 63; tripartite motif containing 63, E3 ubiquitin protein ligase, SLN; sarcolipin, CAPN1; calpain 1, large subunit, MYOD1; myogenic differentiation 1, FBX032; F-box protein 32, CAPN2; calpain 2, large subunit, FST; follistatin, pre-ex; pre-exercise, post-ex; post-exercise. * Significantly (p≤0.05) different from corresponding pre-exercise value. # Significantly different from young. Data bars are mean ± SEM.

Young and older adults performed a single bout of unaccustomed resistance exercise (8 sets, 10 repetitions at 70% of 1-repetition maximum weight). Muscle biopsies were collected before exercise and 15 hours after exercise to measure the expression of selected canonical genes involved in muscle protein turnover by RT-PCR (Figure 3). Myostatin (MSTN), a negative regulator of muscle growth and development, decreased following exercise in young, but not old adults (Figure 3A). Similarly, MyoD, which is a positive regulator of muscle differentiation in response to exercise, increased significantly in young following exercise, but not in old (Figure 3B). TRIM63, which encodes an E3 ubiquitin ligase known as muscle RING Finger 1, decreased significantly after exercise in young but not older adults (Figure 3D). Another ubiquitin ligase, FBXO32, was robustly increased after exercise in young but not old (Figure 3F). Calpain 1 and 2, calcium-activated neutral proteases, were either decreased (CAPN1, Figure 3H) or increased (CAPN2, Figure 3I) following exercise in young but not old. FOXO3, a transcription factor that triggers apoptosis was similarly decreased with exercise in young and old (Figure 3G). Taken together, the mRNA expression of several genes involved in regulating muscle protein turnover exhibit attenuated changes in response to exercise in older adults compared to young. Of note, there were age differences in the baseline, preexercise expression of many of these genes (MSTN, FST, TRIM63, SLN, FBXO32, CAPN1, CAPN2) consistent with a state of active turnover in muscle of older adults before exercise.

### *In vivo* measurements of muscle protein synthesis reveal similar anabolic responses in young and old

**Figure 4:**
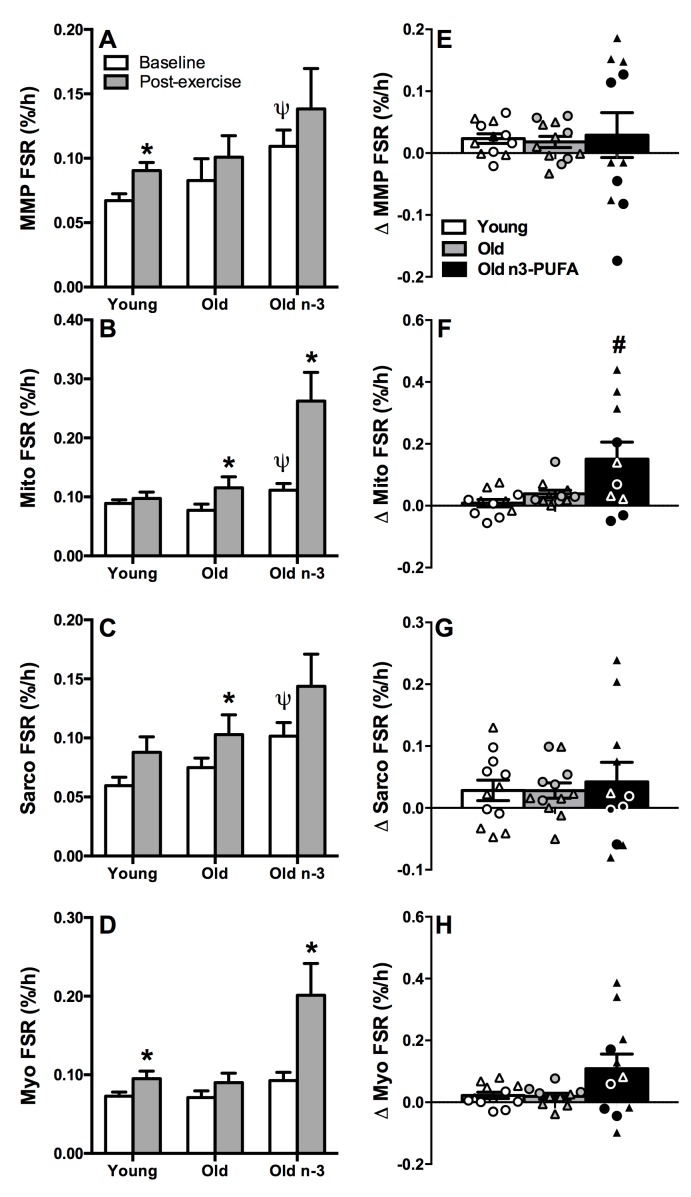
Postabsorptive and exercise-stimulated muscle protein synthesis Muscle protein synthesis was measured from the rate of incorporation of isotopically labeled amino acid into muscle proteins. Protein synthesis was measured at baseline (postabsorptive) and 15-18 hours following a single bout of exercise for mixed muscle (MMP) (**A, E**), mitochondrial fraction (Mito) (**B, F**), sarcoplasmic fraction (Sarco) (**C, G**), and myofibrillar fraction (Myo) (**D, H**). Postabsorptive protein synthesis was similar in young and old in all fractions. Mixed muscle and myofibrillar protein synthesis increased with exercise in young but not old. Mitochondrial and sarcoplasmic protein synthesis increased with exercise in old but not young. n3-PUFA supplementation increased mitochondrial and myofibrillar protein synthesis after exercise. The change in FSR (Δ=post exercise-baseline) is shown in the right panel (**E-H**), illustrating responders and non-responders. * Significantly (p≤0.05) different from corresponding baseline value. Ψ Significantly different from corresponding pre-intervention value. # Significantly different from young. Data bars are mean ± SEM. Circles denote men and triangles denote women.

Skeletal muscle protein synthesis rates were measured by intravenous administration of isotopically labeled phenylalanine ([ring-^13^C_6_]phenylalanine) and subsequent measurement of the rate of incorporation of the labeled amino acid into muscle protein pools by mass spectrometry. Baseline postabsorptive fractional synthesis rates (FSR) were similar in young and older adults when measured in mixed muscle pool (Figure 4A), mitochondrial fraction (Figure 4B), sarcoplasmic fraction (Figure 4C), and myofibrillar fraction (Figure 4D). Protein synthesis was measured again 15-18 hours following the single bout of resistance exercise to determine if the aforementioned gene expression changes with age were accompanied by direct evidence of anabolic resistance (i.e., the translational rate of muscle proteins). Mixed muscle FSR increased significantly after exercise in young adults (Figure 4A). Although the increase in mixed muscle FSR with exercise did not reach statistical significance in older adults, we did not observe any significant differences when we compared the change in FSR from rest to post-exercise in young and old (Figure 4E). Mitochondrial and sarcoplasmic FSR increased significantly in older, but not young adults following exercise (Figure 4B, C) but did not differ in the change in FSR from rest to post-exercise (Figure 4F, G). Similar to mixed muscle FSR, myofibrillar FSR increased significantly after exercise in young but not older adults (Figure 4D), but we did not observe any significant differences when we compared the change in FSR from rest to post-exercise in young and old (Figure 4H). Altogether, the baseline and post-exercise measurements of mixed muscle FSR and subcellular fractions did not reveal any age-related impairments in postabsorptive muscle protein synthesis or anabolic responsiveness.

### n3-PUFA increase postabsorptive protein synthesis and anabolic response to exercise in older adults

The measurements of postabsorptive and post-exercise muscle protein synthesis were repeated in older adults following 16 weeks of daily supplementation (3.9g/day) of n3-PUFA. There was a significant increase in postabsorptive mitochondrial (Figure 4B) and sarcoplasmic (Figure 4C) protein synthesis in older adults following n3-PUFA supplementation. Small but non-significant increases were also observed for mixed muscle (Figure 4A) and myofibrillar (Figure 4D) fractions in the basal condition. After exercise, FSR of mitochondrial and myofibrillar fractions were significantly higher than postabsorptive FSR (Figure 4B, D) with increases in MMP and sarcoplasmic fractions that did not reach statistical significance. The exercise-induced increment in FSR in each fraction was compared in a pairwise manner in older adults before and after the n3-PUFA intervention (Figure 4 E, F, G, H). There was notable heterogeneity in the response as indicated by the individual data points showing that some individuals exhibited robust increases in exercise-stimulated protein synthesis whereas others demonstrated little improvement. The exercise stimulated increase in mitochondrial FSR was significantly greater in older adults following n3-PUFA supplementation compared to young (Figure 4F). A similar trend (P=0.07) was observed for myofibrillar FSR (Figure 4H). In the mixed muscle and sarcoplasmic fractions, n3-PUFA enhanced exercise stimulated protein synthesis in only a fraction of the individuals, with others showing no change or even a decrease compared with pre-intervention values (Figure 4A, C).

### Skeletal muscle whole-transcriptome sequencing

**Figure 5:**
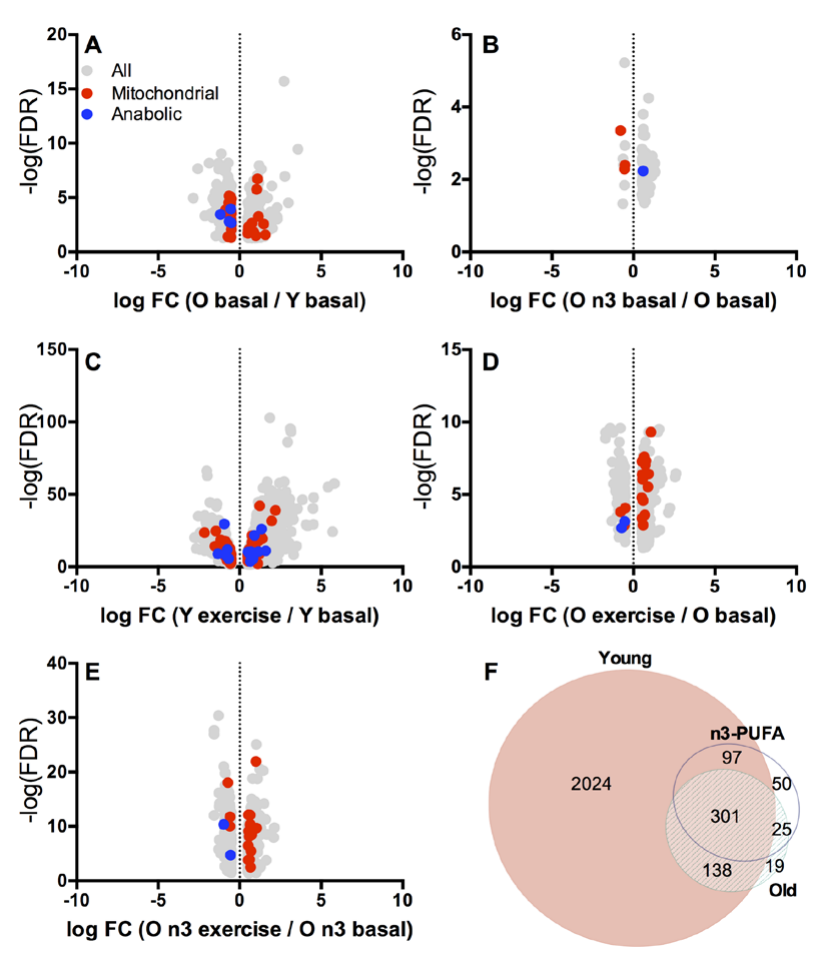
Whole muscle transcriptomics Transcriptional patterns in skeletal muscle were evaluated by RNA sequencing. Volcano plots (**A-E**) show log fold change (FC) vs. –log false discovery rate (FDR) for transcripts that were significantly (p≤0.05) different between young and old at baseline (**A**), old before and after intervention at baseline (**B**), young before and after exercise (**C**), old before and after exercise (**D**), and old before and after exercise following n3-PUFA supplementation (**E**). Downregulated genes are on the negative x axis while upregulated genes are on the positive x axis. Mitochondrial related genes are red and genes involved in muscle protein metabolism are blue. The Venn Diagram (**F**) shows transcripts (Supplemental Table 2) that were differentially expressed with exercise in Young, Old (pre-intervention) and Old n-3 (post-intervention).

To gain mechanistic insights into the effects of aging, exercise and n3-PUFA on skeletal muscle function, next-generation sequencing was performed using RNA extracted from muscle biopsy specimens collected before exercise and 15 hours following exercise. A total of 834 genes were significantly different between young and old at baseline in the postabsorptive state (Figure 5A). Of these genes, 39 were classified as mitochondria-related genes (14 upregulated with age, 25 downregulated with age). A comparison of postabsorptive biopsies from old individuals before and after n3-PUFA intervention (Figure 5B) revealed that a total of 63 genes were significantly changed following n3-PUFA (53 upregulated following n3-PUFA, 10 downregulated). Of these genes, 3 were classified as mitochondria-related genes, all of which were downregulated by n3-PUFA. The heatmaps contained in Supplemental Figures 1-6 show fold changes in reference to the young group in older adults before and after n3-PUFA supplementation for selected gene groups. Genes related to oxidative phosphorylation (Supplemental Figure 1) and the tricarboxylic acid (TCA) cycle (Supplemental Figure 2) display a general pattern where older adults exhibit decreased abundance of the majority of these genes compared to young and further decreases following n3-PUFA.

Next, we examined the genes that were significantly up- or downregulated in response to the single bout of exercise in young and older adults. A total of 2,560 genes were significantly changed in response to exercise in young participants (Figure 5C). Of these genes, 97 were classified as mitochondria-related genes (51 upregulated and 46 downregulated with exercise). We manually curated a list of genes known to be involved in regulating muscle protein turnover of which we found 17 to be significantly influenced by exercise in young (12 upregulated, 5 downregulated). Gene expression fold changes with exercise in selected gene categories are given in Supplemental Figures 7-13 (Oxidative phosphorylation, TCA cycle, Lipid oxidation, ROS production, Glycolysis, Anabolic response, and Inflammation). In older adults, there were far fewer genes (483) significantly changed with exercise (Figure 5D). A total of 17 mitochondrial-related genes were altered by exercise in older adults with 14 upregulated and 3 downregulated. Only 15 genes involved in protein turnover were changed in response to exercise in the older group (9 upregulated, 6 downregulated). Following 16 weeks of n3-PUFA supplementation, a similar number of genes (473) were significantly changed in response to exercise in older adults compared to the pre-intervention measurement (Figure 5E). Mitochondrial genes numbered 17, with 14 upregulated and 3 downregulated with exercise. There were 2 genes involved in protein turnover that were significantly altered (both downregulated with exercise) following n3-PUFA supplementation.

The Venn diagram in Figure 5F shows the genes that were significantly up or downregulated in response to exercise in young, old before n3-PUFA and old following n3-PUFA. The single bout of exercise altered 2,560 genes in young but only 483 genes in the older adults before the intervention. There were 439 genes in common between young and old. Following 16 weeks of n3-PUFA supplementation in older adults, there were 473 genes significantly altered in response to exercise. Of these genes, 326 were in common with pre-intervention gene responses, 398 were in common with young gene responses to exercise, and 50 were unique. We interrogated these 50 unique genes in an attempt to identify potential mechanisms that could explain the significant reduction of mitochondrial ROS production, increase in postabsorptive muscle protein synthesis, and potentiation of anabolic response to a single bout of exercise. Notably, there was downregulation of several genes known to be negative regulators of muscle growth and proliferation (*dusp1, frzb, hbp1*), downregulation of atrophy-related genes (*bmp6*), and upregulation of genes that promote protein synthesis (*mrps12, spon2, prox1, nos1*) and protein folding/assembly (*fkbp2, sptbn4*). Furthermore, there was downregulation of inflammation-related genes (mmd, cxcl9) and upregulation of the adiponectic paralig *c1qtnf6*, which modulates inflammation by countering the actions of TGF beta. Of the 9 candidate genes that were interrogated by RT-PCR, myostatin, a negative regulator of muscle anabolism, exhibited significant downregulation with exercise following n3-PUFA supplementation, which was not apparent before the intervention (Figure 3A). In sum, RNA sequencing revealed a transcriptional signature that is consistent with the observed mitochondrial phenotype in skeletal muscle from older adults. Furthermore, the transcriptional changes induced by a single bout of exercise were attenuated in older adults compared to young with subtle effects of n3-PUFA on baseline muscle gene expression or gene expression changes following a single bout of exercise. However, the expression of small number of genes that regulate muscle protein turnover and inflammation were uniquely altered by exercise in older adults only after 16 weeks of n3-PUFA supplementation.

## DISCUSSION

The major salient observations in these studies are related to the effects of age and n3-PUFA supplementation on mitochondrial physiology, protein metabolism, and gene expression in human skeletal muscle. First, unlike our previous rodent experiments [43, 44], we find that n3-PUFA did not increase mitochondrial oxidative capacity in older adults. However, n3-PUFA supplementation reduced mitochondrial oxidant emission rates. Second, we find no age-related impairments in muscle protein synthesis rates at baseline or following exercise in spite of clear attenuation of transcriptional response to exercise compared to young. Finally, 16 weeks of n3-PUFA supplementation increased postabsorptive muscle protein synthesis (particularly mitochondrial and sarcoplasmic fractions) and increased the synthesis rates of mitochondrial and myofibrillar proteins following exercise.

The precedent literature is mixed concerning the effects of aging on muscle protein synthesis under postabsorptive conditions with some investigators reporting reductions in FSR with age [45, 46], while others do not [47, 48]. In the current cohort of healthy older adults, we do not observe any difference in postabsorptive muscle protein synthesis. There is convincing evidence that anabolic responses to nutrition or exercise stimuli are blunted with aging [24-26]. Although we find transcriptional evidence to support the concept of age-related anabolic resistance, the older individuals exhibited remarkably preserved muscle protein synthesis in response to exercise when compared to the young group. One interpretation is that these older adults are somehow able to mount an anabolic response of translational machinery in the face of attenuated transcriptional messages. Another possibility is that the timing of the post-exercise protein synthesis measurements may have missed the point in time where young and old differ in synthesis rate. We chose to measure FSR between 15 and 18 hours following the single bout of exercise based on a previous report that mixed muscle FSR in young and older adults reached peak values 6hr post-exercise and maintained until at least 24 hours post-exercise when using the same exercise paradigm that we employed here [24]. Therefore, the 15-18 hour post-exercise window should capture peak muscle protein synthesis in both age groups. Indeed, we observe a similar increment in muscle protein synthesis rates at 15-18 hours post-exercise as Fry and colleagues observed at 6 hours and 24 hours post-exercise [24].

A major goal of this study was to determine the influence of n3-PUFA on mitochondrial physiology in human skeletal muscle. We recently reported that the anti-inflammatory effects of n3-PUFA were associated with upregulated transcriptional regulators of mitochondrial biogenesis [44] and marked improvements in mitochondrial function in old mice [43]. These early observations in mice fueled the current study to determine if such mitochondrial adaptations were evident in older humans. In contrast to our previous observations in mice, 16 weeks of high dose n3-PUFA did not improve mitochondrial capacity in older humans (Figure 2), nor did we observe any increase in mRNA levels of genes encoding proteins involved in oxidative phosphorylation (Supplemental Figure 1), TCA cycle (Supplemental Figure 2), or lipid metabolism (Supplemental Figure 3). This finding is consistent with our previous study in overweight insulin-resistant individuals where n3-PUFA supplementation did not alter muscle mitochondrial energetics [49]. Similarly, a recent study by Herbst et al. found that 12 weeks of n3-PUFA supplementation increased mitochondrial sensitivity to ADP without increasing maximal state 3 respiration [50]. We did not observe any changes in the respiratory control ratio (State 3/state 4) or ADP:O ratio that would indicate any change in bioenergetic efficiency of mitochondria.

Mitochondrial oxidant emission is another parameter that helps define mitochondrial health. Although no age-related increase in mitochondrial ROS production was apparent in this study, we found that ROS emissions were significantly lower in older adults following n3-PUFA administration. Although polyunsaturated fatty acids have been proposed to have antioxidant properties [51], we are unaware of any precedent literature showing that these bioactive lipids have direct influence on oxidant emitting potential of mitochondria. We do not know the mechanism by which n3-PUFA reduce mitochondrial ROS production, but the finding that n3-PUFA reduce ROS production in older adults is important from the standpoint of muscle health with aging. Aging is often accompanied by increased oxidative stress and accumulation of damaged protein in skeletal muscle [52-54]. Discovering safe and effective ways to preserve the quality and function of the muscle proteome will help forestall many age-related derangements in skeletal muscle. In support of this, we previously found that n3-PUFA decreased post-translational modification of proteins in muscle of old mice [43]; a finding that is likely linked with the effects of n3-PUFA on ROS production and ROS scavenging by endogenous antioxidants.

The potential anabolic effects of n3-PUFA are suggested from observations that n3-PUFA supplementation augments the gains in muscle strength in response to training in elderly women [37]. Further support comes from a series of papers by Smith and colleagues demonstrating that 8 weeks of n3-PUFA supplementation increases protein synthesis rates during a hyperaminoacidemic-hyperinsulinemic clamp in young, middle-aged, and older adults [35, 55]. Motivated by these early studies, we determined if n3-PUFA could potentiate the anabolic responses to a single bout of exercise in older adults. In agreement with Smith et al. [35], we found no significant change in mixed muscle protein synthesis rates in the basal postabsorptive state in older adults following n3-PUFA. However, when we examined protein synthesis rates in subcellular pools, we found that n3-PUFA significantly increased FSR in the mitochondrial and sarcoplasmic pools. Omega-3 fatty acids also potentiated muscle protein synthesis after a single bout of exercise, primarily in the mitochondrial and myofibrillar fractions. These results, combined with previous evidence that n3-PUFA enhance anabolic response to insulin and amino acids, suggest that n3-PUFA increase muscle anabolic responses to nutrition and physical activity in older adults. A recent paper [56] used a very similar experimental design in young healthy men who were given 5g/day fish oil for 8 weeks. Muscle protein synthesis was measured at rest, after a bolus of dietary protein, and after a single bout of exercise chased with a bolus of protein. There were no effects of n3-PUFA on muscle protein synthesis in these young men, which is in contrast to our findings here in older men and women. Key differences between the study of mcGlory et al. and the present study are the timing of post-exercise biopsies, the addition of dietary protein prior to exercise, and subject characteristics, all of which make it difficult to extrapolate data or conclusions between the two studies. It is important to highlight that we observed clusters of responders and non-responders whereby some individuals exhibited robust increases in exercise-stimulated protein synthesis while others do not. Attempts to link responders and non responders with any measured characteristic, biochemical marker, or phenotype did not reveal any clear discriminating factors, but the current study is limited by a small sample size for this type of exploratory analysis. Notably, the high responders observed in figure 4 were largely women. This observation is consistent with a recent study where n3-PUFA improved skeletal muscle function and quality in older women but not older men [38]. Further studies in larger cohorts with placebo control are required to further investigate the possibility that the anabolic effects of n3-PUFA differ between men and women.

In an attempt to identify the mechanisms by which n3-PUFA increased postabsorptive and exercise-stimulated protein synthesis, we first used RT-PCR to evaluate the expression of several genes known to regulate muscle protein turnover in response to exercise (Figure 3). Of the 9 *a priori* identified genes, only myostatin emerged as a gene whose expression in response to exercise was altered by n3-PUFA. As a negative regulator of muscle growth and differentiation, the significantly lower post-exercise myostatin levels following n3-PUFA may partly explain the enhanced anabolic response to exercise. We then mined the RNA sequencing dataset for genes known to regulate muscle protein turnover (Supplemental Figure 12). As a whole, there are no obvious shifts in the gene set, but several individual genes exhibited greater changes following n3-PUFA. These genes included myostatin (*mstn*), the forkhead transcription factor *foxo1* which mediates muscle wasting through control of the expression of several E3 ligases, and calcineurin A (*ppp3ca*) which is a crucial signaling intermediate in muscle fiber hypertrophy. Next, we identified the genes that were uniquely altered with exercise following n3-PUFA supplementation, but not before the intervention (Figure 5F). Several negative regulators of muscle growth and proliferation (*dusp1, frzb, hbp1*) were downregulated, one atrophy-related gene (*bmp6*) was downregulated, and several genes that promote protein synthesis (*mrps12, spon2, prox1, nos1*) and protein folding/assembly (*fkbp2, sptbn4*) were upregulated. These findings are in agreement with recent findings by Yoshino et al. [57] who demonstrate that n3-PUFA increased expression of genes related to extracellular matrix organization and decreased genes related to protein breakdown in skeletal muscle of older adults. Altogether, these data suggest that n3-PUFA alter transcriptional patterns in skeletal muscle that enhance muscle protein turnover. Others show that n3-PUFA may enhance anabolic response to exercise by altering the phosphorylation of signaling transduction proteins in muscle such as mTOR and p70s6k [35]. Taken together, the anabolic effects of n3-PUFA are likely mediated by transcriptional changes as well as changes to the activation of anabolic signaling proteins in skeletal muscle.

There is growing evidence that chronic inflammation may influence muscle anabolic responses to nutrition and exercise. The translation efficiency of mRNA is compromised by high levels of inflammatory cytokines [31], and muscle protein synthesis in older adults is inversely related to levels of inflammatory cytokines [32, 33]. The hypothesis that inflammation may contribute to sarcopenia by inhibiting muscle protein anabolism is further supported from studies where reducing inflammation using non-steroidal anti-inflammatory drugs improved postprandial protein synthesis [34] and enhanced muscle mass and strength gains in response to resistance exercise in older humans [36, 37]. Given the well-documented anti-inflammatory effects of n3-PUFA [41, 42], it reasonable to expect that the increases in muscle protein synthesis observed in this study could be consequent to reduced chronic inflammation. However, we found that 16 weeks of high dose n3-PUFA supplementation did not reduce systemic inflammatory markers TNF-ɑ, CRP, or IL-6 (Table 2). This finding is in agreement with others [35, 38] and might be a reflection of the healthy status of our cohort, since patients with a high inflammatory status are more likely to present with improvements in inflammatory markers, metabolic profile and muscle wasting with n-3 PUFA [58-60]. Nevertheless, at the muscle level, there was downregulation of inflammation-related genes (mmd, cxcl9) and upregulation of the adiponectic paralog *c1qtnf6*, which modulates inflammation by countering the actions of TGF beta in older adults (Figure 5F). The possibility that n3-PUFA modulate local tissue inflammation without influencing circulating inflammatory markers requires further exploration.

The main limitation of the study is the open-label design without placebo control. Furthermore, the small sample size prevents adequately-powered *post hoc* explorations of sex differences or to determine what factors may predict responders and non-responders. Participants in this study were included only after satisfying an extensive list of inclusion and exclusion criteria, which limits the external validity of the results, particularly when generalizing to the aging population at large. Among the strengths of the study was the high compliance rate, minimal attrition rate, and pharmacological dose of n-3 PUFA for an adequate time frame. Gold standard methods were used to evaluate primary outcomes, and studies were conducted within the highly controlled environment of the Clinical Research and Trials Unit where participants were admitted for 2 nights following 3 days of standardized meals at the hospital.

In conclusion, we demonstrate that dietary n3-PUFA induce potentially favorable adaptations within skeletal muscle in older adults, including decreased mitochondrial ROS production, increased muscle protein synthesis rates, and enhanced anabolic response to a single bout of exercise. Future studies are needed to determine if these adaptations translate into meaningful improvements in metabolic and physical function in older adults when n3- PUFAs are given over longer periods of time or in combination with an exercise training program.

## MATERIALS AND METHODS

### Research participants

Twelve young (18-35 years) and 12 older (65-85 years) men and women were recruited from the local community and provided written informed consent as approved by the Mayo Foundation Institutional Review Board. The study conformed to the principles outlined in the Declaration of Helsinki. Participants were excluded if they consumed n-3 PUFA supplements within 3 months of the study, or if they participated in structured exercise (>30 min, ≥3 times/week). Participants were also excluded if they had fish or shellfish allergy, anemia (hemoglobin <11 g/dL for women and <12 g/dL for men), diabetes (or fasting blood glucose ≥126 mg/dL), cardiovascular disease, liver, renal or untreated thyroid disease, and any debilitating musculoskeletal or pulmonary disease which would impede participation in the exercise portion of the study. Pregnancy, breastfeeding, smoking and other substance abuse were also excluded, as well as medications which affect muscle metabolism (beta blockers, corticosteroids, tricyclic antidepressants, benzodiazepines, opiates, barbiturates) and anticoagulants. We did not exclude for statins or oral estrogens. All participants completed a screening visit followed by a two-day inpatient study to evaluate muscle protein synthesis, acute exercise responsiveness, and mitochondrial physiology. Older participants were studied again following a 4-month open-label intervention of dietary n3-PUFA (3.9g/day). The young participants were studied as a comparison group to determine the extent of skeletal muscle dysfunction with aging and did not undergo any intervention. The study design is illustrated in Figure 1.

### Screening

Eligible participants were identified using a screening questionnaire via telephone or email. Those who met eligibility criteria reported to the Clinical Research and Trials Unit (CRTU) at Mayo Clinic Hospital, St. Marys Campus after fasting from 22:00 hrs the night before to assess eligibility by physical examination, resting electrocardiogram and comprehensive blood tests including complete blood count and biochemical tests of glucose, insulin, Hba1c, liver (ALT, AST, bilirubin), kidney (creatinine, GFR) and thyroid (TSH) function, as well as coagulation (INR), and lipid panel (Total, LDL, HDL-cholesterol, Triglycerides). Participants had a consultation with a dietician to discuss food preferences and underwent measurements of body composition by dual-energy X-ray absorptiometry (DEXA) to determine whole body fat mass, body fat, and fat free mass (FFM) (Lunar DPX-; Lunar Radiation, Maddison, WI).

### VO_2_ peak

Whole-body peak oxygen uptake (VO_2_ peak) was measured by indirect calorimetry on a stationary cycle ergometer with an incremental workload as previously described [49]. The VO_2_ peak protocol began at a workload of 50 Watts for young adults and old males or 25 Watts for old females, with an increase of 30 Watts every 2 min for young males or 20 Watts for young females and older adults. Participants were monitored throughout rest, exercise, and recovery by 12 lead ECG, blood pressure, and expired gasses. Subjective level of exhaustion was assessed using the Borg scale.

### 1 Repetition Maximum (1RM)

Maximal knee extensor strength of the left leg was evaluated on two separate occasions under the guidance of the investigators. The protocol included a warm-up set of 10 repetitions using a light weight, followed by 2 sets of up to 10 repetitions with 3 min-interval between sets. 1 RM was calculated from the average of the two sets using the formula: 1RM = ω·(1+r/30), where ω is the weight in arbitrary units and r the number of repetitions per set.

### Inpatient study design

At least 7 days after the VO_2_ peak test, participants were admitted to the CRTU. For three days prior to admission they were provided a weight-maintaining diet by the CRTU metabolic kitchen. Macronutrient distribution of the diet was 20% protein, 50% carbohydrate and 30% fat. On the evening of the 3^rd^ day of the diet, participants were admitted to the CRTU at 1700 hrs and consumed nothing but water after 2200 hrs. At 0500 hrs, a primed (1.5 mg·kg FFM^−1^), continuous infusion (1.5 mg·kg FFM^−1^·hr^−1^) of ^13^C_6_-Phenylalanine was initiated as shown in Figure 1B. Resting energy expenditure (REE) was measured for 20 min starting at 0630 hrs using a ventilated hood and indirect calorimetry [49]. Muscle biopsies were obtained from the right *vastus lateralis* at 0800 hrs and 1100 hrs for measuring post-absorptive protein synthesis rates from incorporation of intravenous labeled amino acid into muscle protein pools. Biopsies were collected under local anesthesia (2% lidocaine) using a modified Bergstrom needle. Following the biopsy at 1200 hrs, participants were given a standardized meal containing 10 kcal/kg of 20% protein, 50% carbohydrate, and 30% fat. At 1600 hrs, participants performed seated unilateral leg extension using only the left leg. Following a warm-up set, subjects completed 8 sets of 10 repetitions at 70% of their 1 RM, determined during the outpatient visit. Three minutes of rest were given between sets. This exercise paradigm has been previously used to assess the effects of acute resistance exercise on muscle anabolic response with aging [24]. The exercise was supervised by a member of the study team at the Dan Abraham Healthy Living Center, Saint Mary's Hospital, where participants were escorted with the use of a wheelchair. A second meal was given at 1800 hrs with caloric content to achieve weight maintenance, after which participants remained fasted until the second set of protein synthesis measurements on day 2. The tracer infusion was initiated at 0400 hrs, followed by two serial muscle biopsies on the exercised leg at 0700 and 1000 hrs. Participants were offered a meal and were discharged by noon of the second day.

### n-3 PUFA intervention

The n3-PUFA softgels were supplied by Sancilio and Company, Inc. (Riviera Beach, FL) and stored in the Mayo Clinic Research Pharmacy at room temperature. During the intervention phase of the study, participants were instructed to swallow 2 softgels twice per day with meals. The n3-PUFA softgels contained 675 mg of EPA and 300 mg of DHA for a total daily dosage of 3.9 g/day and ratio of 2.25 EPA:DHA. The total omega-3 fatty acids content of each capsule was 1050 mg, which also included 75 mg of “non n3-PUFA” omega-3 fatty acids. The total fat content of the capsule was 1200 mg. This dose of n3-PUFA was chosen because it has been shown to augment the anabolic response of skeletal muscle under hyperinsulinemia and high amino acids [55]. Every 4 weeks, participants returned to the CRTU to pick up a new prescription, returned any remaining capsules, and give a fasting blood sample. The Mayo Clinic Research Pharmacy maintained records of receipt, dispensation, and return pill counts for compliance. Compliance was also assessed at the end of the study by monthly measurements of EPA and DHA content of red blood cells (RBC), as well as plasma levels of EPA and DHA measured by mass spectrometry. The duration of the intervention was 4 months.

### Muscle mitochondria function

#### Mitochondrial oxidative capacity

Approximately 60 mg of fresh muscle tissue was homogenized on ice immediately after the first muscle biopsy on day 1. Mitochondria were isolated by differential centrifugation and suspended in a respiration buffer (MiR05; Oroboros, Innsbruck, Austria) as previously described [61]. Respiration of isolated mitochondria was measured by high resolution respirometry (Oxygraph 2K, Oroboros Instruments, Innsbruck, Austria) using a stepwise protocol to evaluate various components of the electron transport system [49, 53]. Oxygen flux rates (*JO_2_*) were assed in the presence of carbohydrate (glutamate + malate) and lipid (palmitoyl-L-carnitine) substrates [61]. Mitochondrial protein content was measured using a colorimetric assay (Pierce 660-nm Protein Assay). Oxygen consumption was expressed to tissue wet weight and also normalized to mitochondrial protein content. Mitochondrial coupling efficiency was measured from the respiratory control ratio (RCR) calculated by the quotient of state 3 and state 4 respiration. ADP:O ratio was determined by dividing the amount of ADP added to the chamber to the amount of atomic oxygen used during state III respiration, and by using PRISM v6.0e software (GraphPad Software Inc, La Jolla, CA).

#### Mitochondrial reactive oxygen species (ROS) production

Hydrogen peroxide production in isolated mitochondria (*mt*H_2_O_2_) was measured with a Fluorolog 3 spectrofluorometer (HORIBA Jobin Yvon) by continuously monitoring of oxidation of Amplex Red under the same respiratory conditions as described for oxygen consumption measurements as we have previously described [44, 53]. The fluorescent signal was adjusted for background auto-oxidation and calibrated to a standard curve. Rates of H_2_O_2_ production were normalized to tissue wet weight and to protein content of the mitochondrial isolate.

### Muscle protein synthesis rates

Isotopic enrichment in muscle tissue fluid and muscle protein pools were measured by HPLC and tandem mass spectrometry (MS/MS) as previously described [44, 53, 62, 63]. Briefly, total mixed muscle, myofibrillar, mitochondrial, and sarcoplasmic (cytosolic), as well as tissue fluid free amino acid fractions were isolated from 10 mg of quadriceps. The protein was hydrolyzed overnight in 6N HCL at 110°C. Phenylalanine from both tissue fluid and hydrolyzed proteins were isolated further via cation exchange column-AG50 and dried down prior to derivatizating to isobutyl esters. Data acquisition was performed in positive electrospray ionization mode with selecting ion monitoring at 222.4 > 121.6 and 226.4 > 125.6 for the m+2 and m+6 fragments of phenylalanine and L-[ring ^13^C_6_]-phenylalanine respectively. Moles percent enrichment (MPE) was calculated against a 6-point-enrichment standard curve. Fractional synthesis rate of mixed muscle, myofibrillar, mitochondrial and sarcoplasmic proteins was calculated from the increment in ^13^C_6_-Phenylalanine enrichment in the two serial muscle biopsies (E_2_-E_1_) using the average enrichment of tissue fluid ^13^C_6_-Phenylalanine as the precursor pool enrichment (Ep), based on the formula: FSR = (E_2_-E_1_) ·100/ Ep · time [%·h^−1^].

### Quantitative PCR

Approximately 20 mg of muscle was powdered in liquid nitrogen and total RNA extracted using a kit (RNeasy Fibrous Tissue, Qiagen) with DNAse treatment. RNA concentration and purity (A260/A280>2.0 for all samples) were determined by spectrophotometry (Nanodrop). Two micrograms of RNA were reverse-transcribed to cDNA according to manufacturer's instructions (Applied Biosystems). Quantitative real-time polymerase chain reaction (qPCR) was performed in 384 well clear plates with 20μl reaction volume using 20ng cDNA. Amplification conditions were 10 minutes at 60°C followed by 40 cycles of denaturing (95°C for 15 s) and annealing (60°C for 60 s) using a ViiA7 thermocycler (Applied Biosystems). Samples were amplified with multiplex conditions in triplicate on a single plate with a ‘no template’ control, internal repeated control, and 7 point relative standard curve spanning 4 log dilutions. Primers and probes were commercially produced (Applied Biosystems, TaqMan Gene Expression Assays) for 9 gene targets as shown in Supplemental Table 1. Efficiencies of the target and reference genes were similar (∼95-100%) from the standard curve. Relative fold change was determined using the 2-ΔΔCt approach [64] with resting biopsy sample as the control condition.

### mRNA sequencing

Total RNA was isolated as described above and RNA libraries were prepared according to the manufacturer's instructions for the TruSeq RNA Sample Prep Kit v2 (Illumina). Libraries were loaded onto paired end flow cells following the standard protocol for the Illumina cBot and cBot Paired end cluster kit version 3. Flow cells were sequenced as 51 × 2 paired end reads on an Illumina HiSeq 2000 using TruSeq SBS sequencing kit version 3 and HCS v2.0.12 data collection software. Base-calling was performed using Illumina's RTA version 1.17.21.3. The RNA-Seq data was analyzed using MAP-RSeq v.1.2.1 [65], the Mayo Bioinformatics Core pipeline. MAP-RSeq consists of alignment with TopHat 2.0.6 [66] against the hg19 genome build and gene counts with the HTSeq software 0.5.3p9 (http://www.huber.embl.de/users/anders/HTSeq/doc/overview.html) using gene annotation files obtained from Illumina (http://cufflinks.cbcb.umd.edu/igenomes.html). Normalization and differential expression analysis were performed using edgeR 2.6.2 [67].

### Analytical methods

Glucose was measured in plasma samples by photometric hexokinase method. Insulin was measured by a two-site immunometric assay using electro chemiluminescence immunoassay “ECLIA” detection (Roche Diagnostics, Indianapolis, IN). HOMA-IR index was calculated by the formula: fasting glucose (mmol/L) x fasting insulin (μIU/mL)/ 22.5 [68]. Interleukin (IL)-6, IL-1β and Tumor necrosis factor alpha (TNF-α) were measured from serum samples using a solid-phase ELISA by the Quantikine High Sensitivity Immunoassay. Leptin and adiponectin were measured by radioimmunoassay, and C-reactive protein by particle enhanced immune-turbidimetric assay. Blood measurements were done in the Mayo Clinic Immunochemical Core Laboratory. Plasma free fatty acids were measured against a standard curve on an Agilent 6460 triple quadrupole liquid chromatography/mass spectrometer (LC/MS) as previously described [69].

### Statistical methodology

Unpaired ttests were used to compare mitochondrial parameters (respiration rates, ROS production) between young and old groups prior to the intervention. Paired ttests were used to examine the effects of n3-PUFA supplementation on these variables in older adults. For variables that were measured at multiple time points such as gene expression and FSR before and after exercise, we identified *a priori* comparisons that would be made using paired ttests (pre- vs. post-exercise in young, pre- vs. post-exercise in old, pre-exercise before vs. after intervention) as well as unpaired comparisons by ttests (baseline young vs. baseline old, post-exercise young vs. post-exercise old). When variables were not normally distributed, the Wilcoxon signed rank test was used for pre-post intervention for the older group, as well as for comparisons pre-post-exercise for both age groups. Continuous variables were summarized using mean ± SD or median (interquartile range, IQR) as appropriate. Statistical analysis was performed using JMP Software (SAS Institute, Cart, NC) and PRISM v6.0e (GraphPad Software Inc, La Jolla, CA). The Venn diagram in Figure 5 was generated using EulerAPE v3 (eulerdiagrams.org).

## SUPPLEMENTARY MATERIALS FIGURES AND TABLES




