# Supplementary material for: Influence of omega-3 fatty acids on skeletal muscle protein metabolism and mitochondrial bioenergetics in older adults
Source: Aging (Albany NY). 2017 Apr 5;9(4):1096–115. doi: 10.18632/aging.101210 (PMC5425117; doi:10.18632/aging.101210)
Supplement: Supplementary file 1 [file aging-09-1096-s001.pdf]

SUPPLEMENTARY MATERIALS

Supplemental Figures

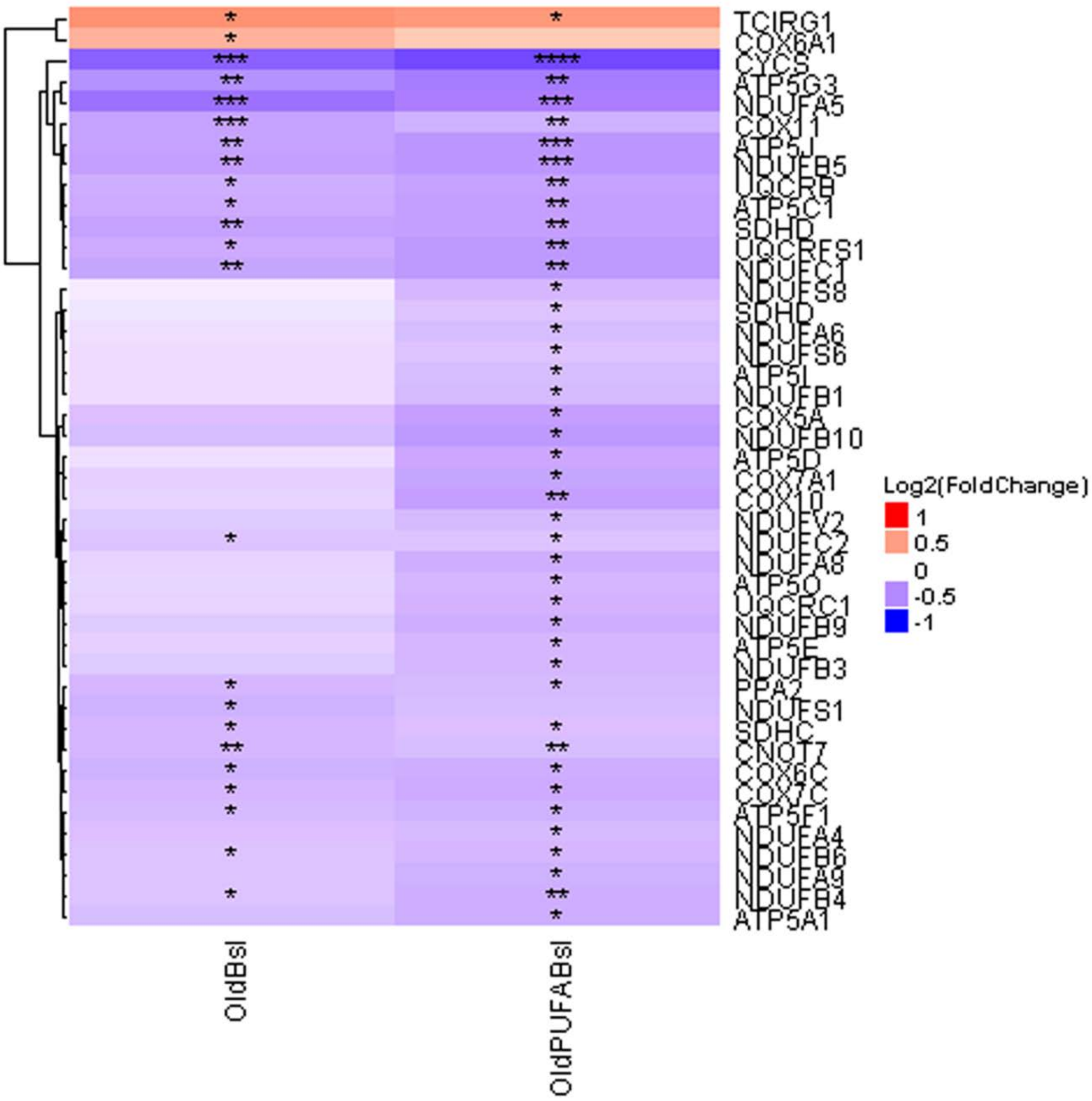

Supplemental Figure 1. The heatmap shows fold changes for genes related to oxidative phosphorylation in old baseline preintervention and old baseline postintervention relative to young baseline. A positive fold change (red) indicates upregulated in old compared to young, and a negative fold change (blue) indicated downregulated in old compared to young. * denotes where the fold change was statistically significant (p≤0.05).

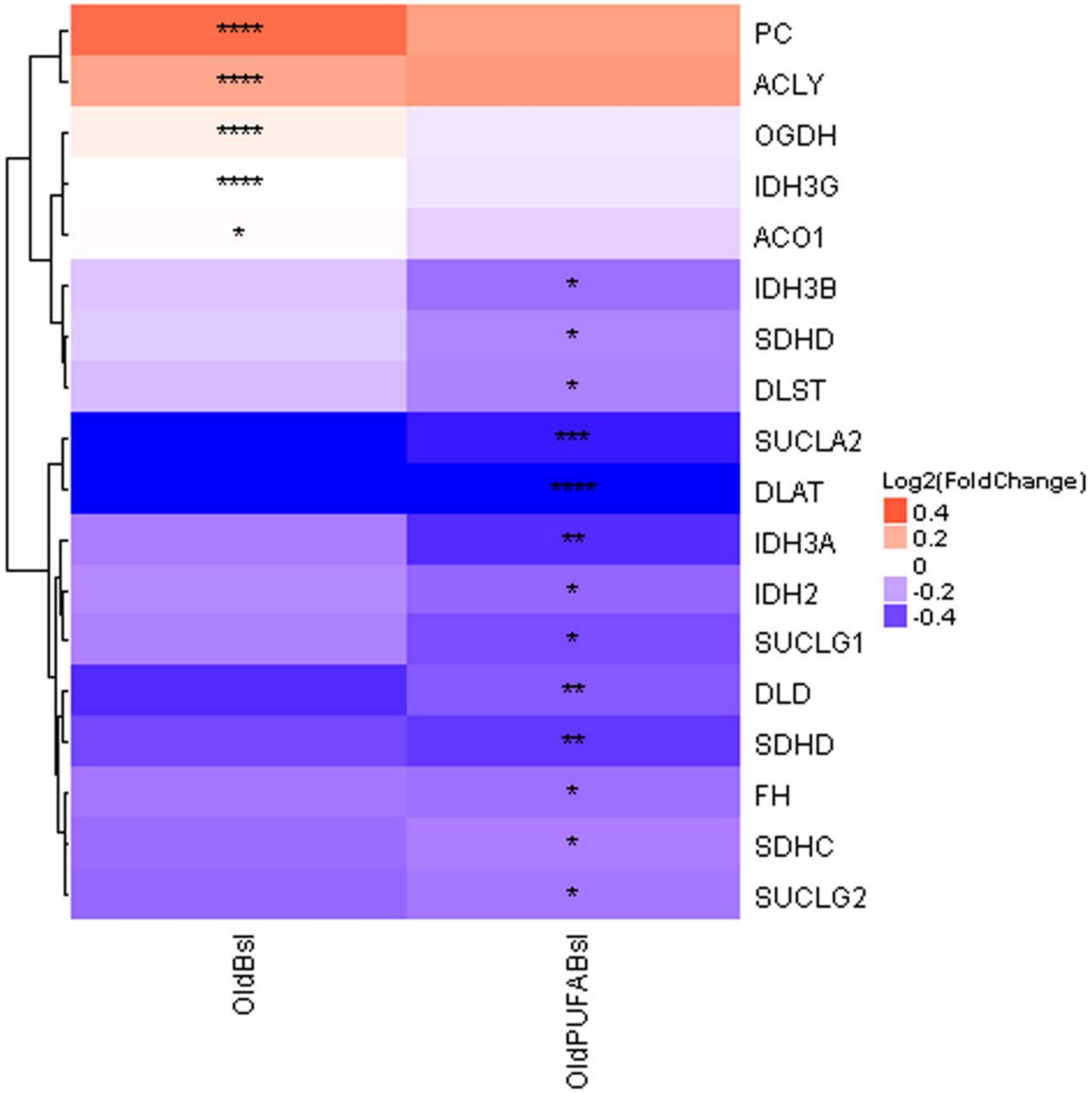

Supplemental Figure 2. The heatmap shows fold changes for genes related to tricarboxylic acid cycle in old baseline preintervention and old baseline postintervention relative to young baseline. A positive fold change (red) indicates upregulated in old compared to young, and a negative fold change (blue) indicated downregulated in old compared to young. * denotes where the fold change was statistically significant (p≤0.05).

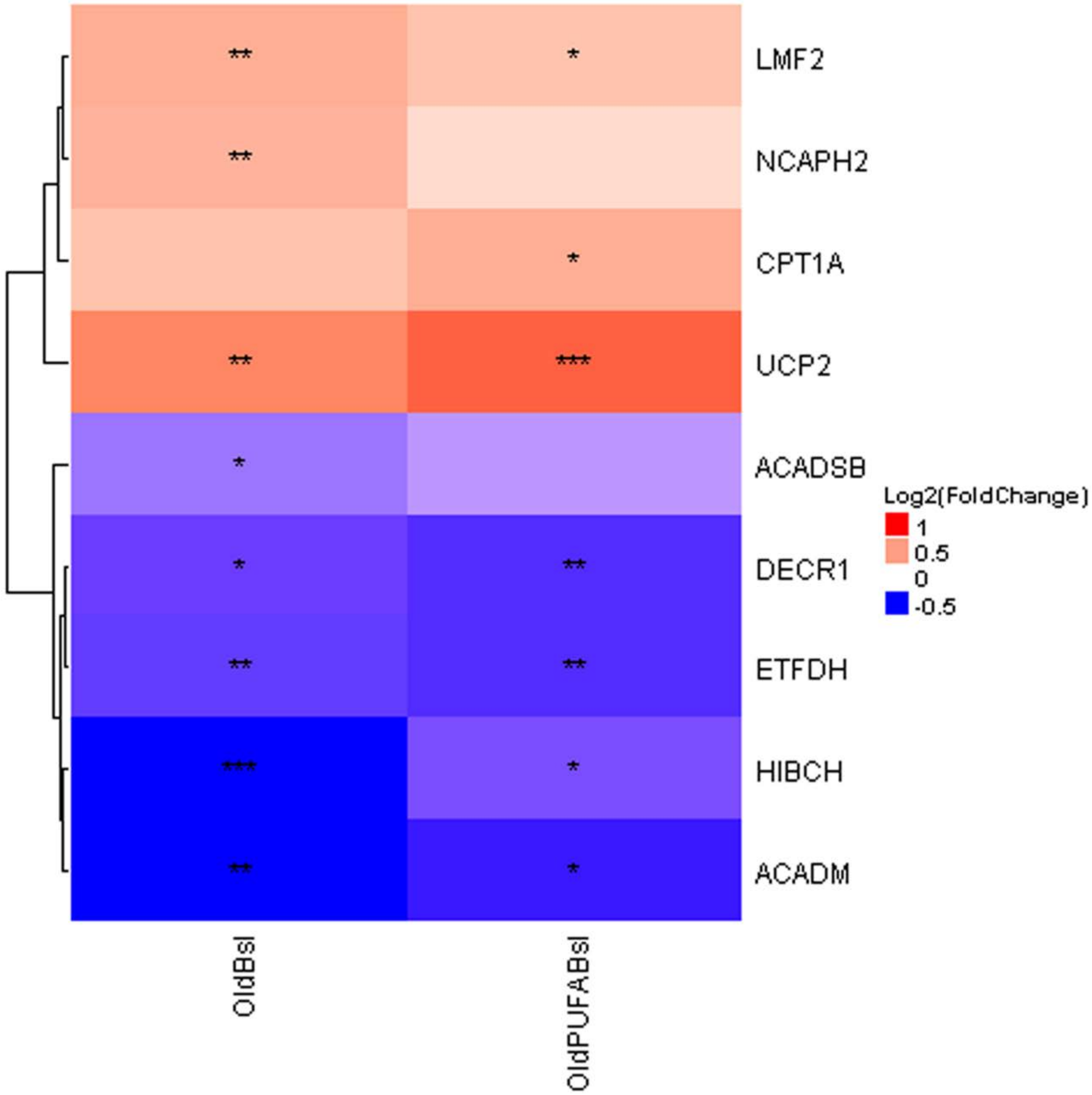

Supplemental Figure 3. The heatmap shows fold changes for genes related to lipid metabolism in old baseline preintervention and old baseline postintervention relative to young baseline. A positive fold change (red) indicates upregulated in old compared to young, and a negative fold change (blue) indicated downregulated in old compared to young. * denotes where the fold change was statistically significant (p≤0.05).

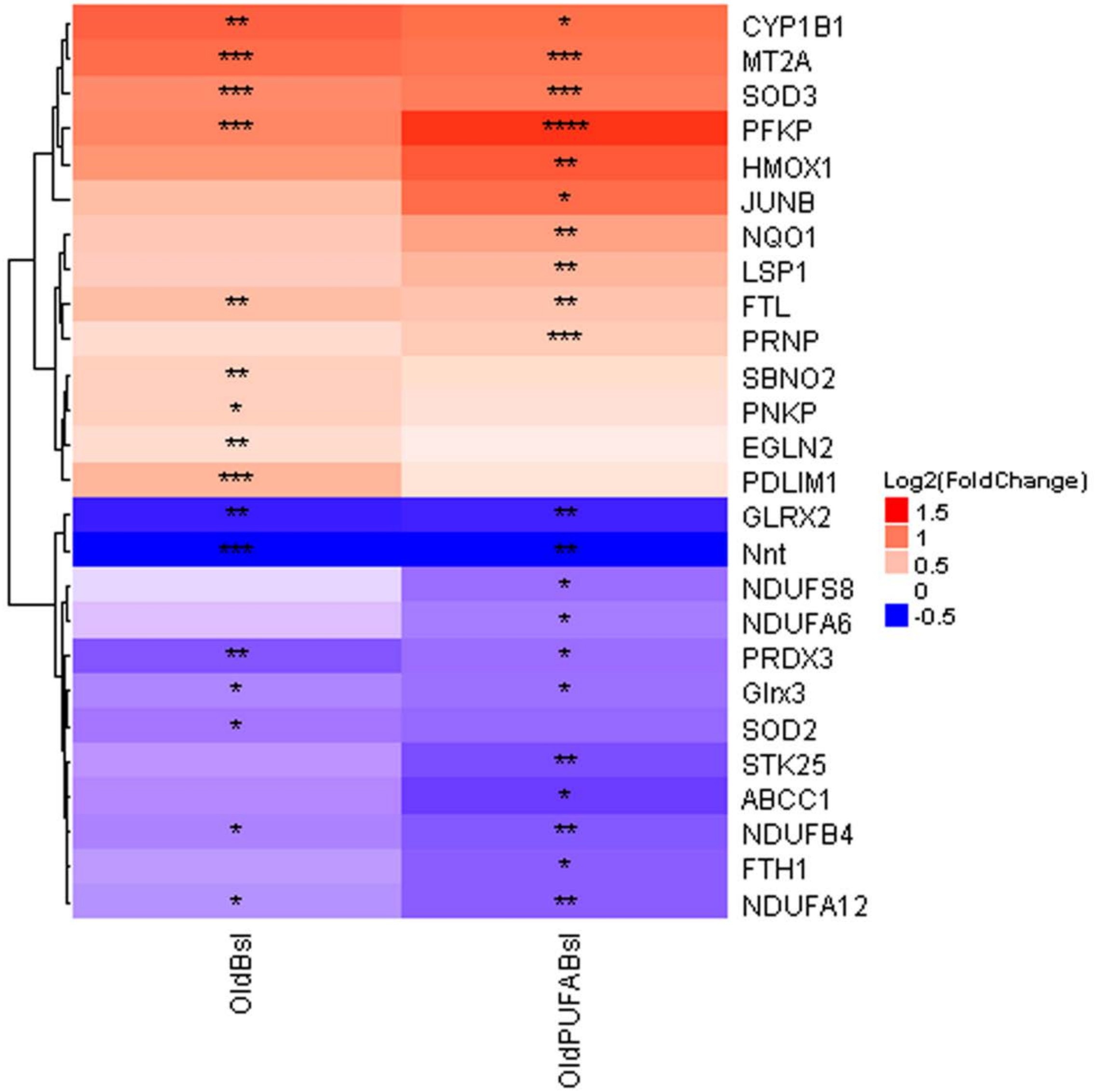

Supplemental Figure 4. The heatmap shows fold changes for genes related to oxidative stress in old baseline preintervention and old baseline postintervention relative to young baseline. A positive fold change (red) indicates upregulated in old compared to young, and a negative fold change (blue) indicated downregulated in old compared to young. * denotes where the fold change was statistically significant (p≤0.05).

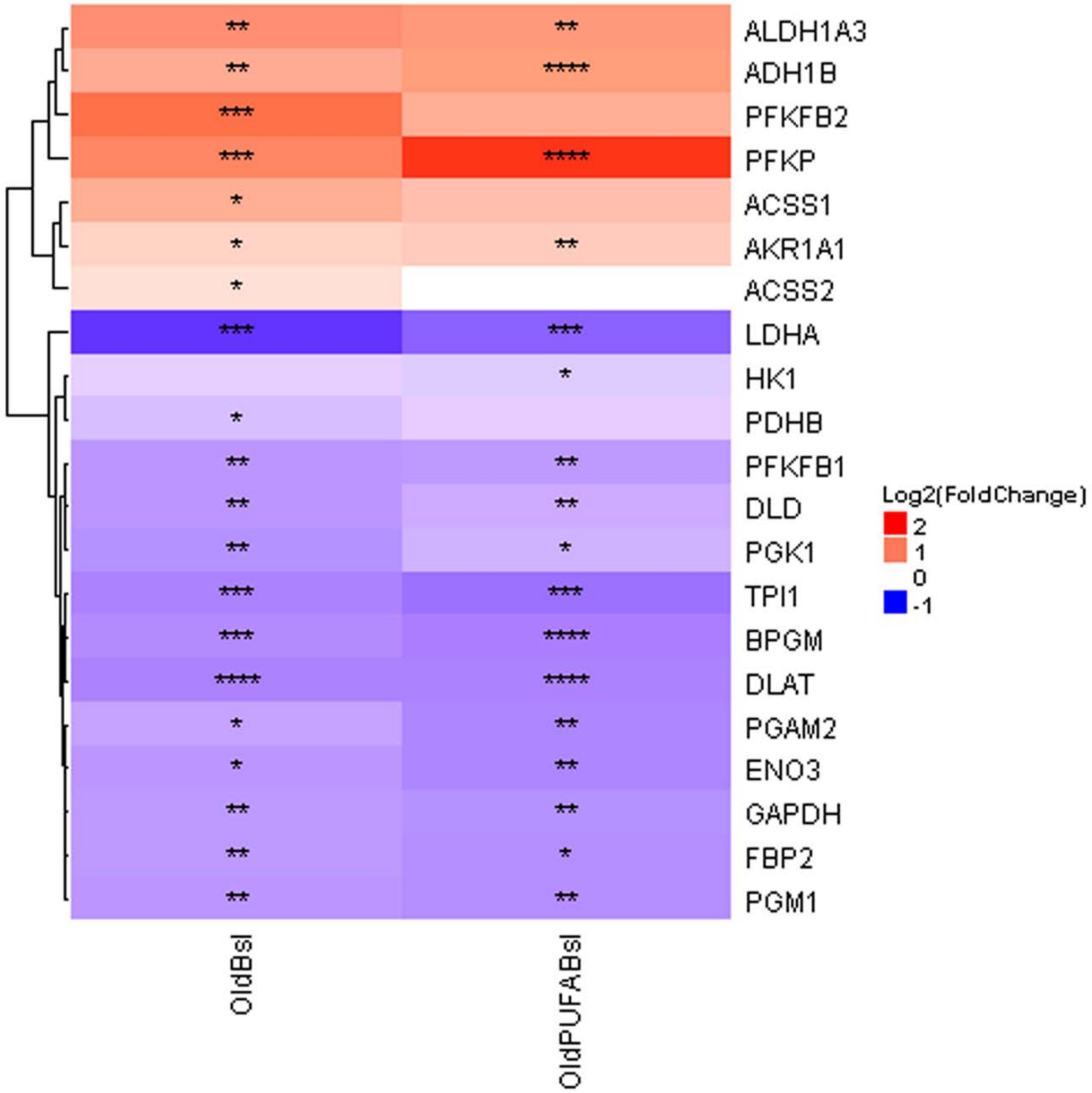

Supplemental Figure 5. The heatmap shows fold changes for genes related to glycolysis in old baseline preintervention and old baseline postintervention relative to young baseline. A positive fold change (red) indicates upregulated in old compared to young, and a negative fold change (blue) indicated downregulated in old compared to young. * denotes where the fold change was statistically significant (p≤0.05).

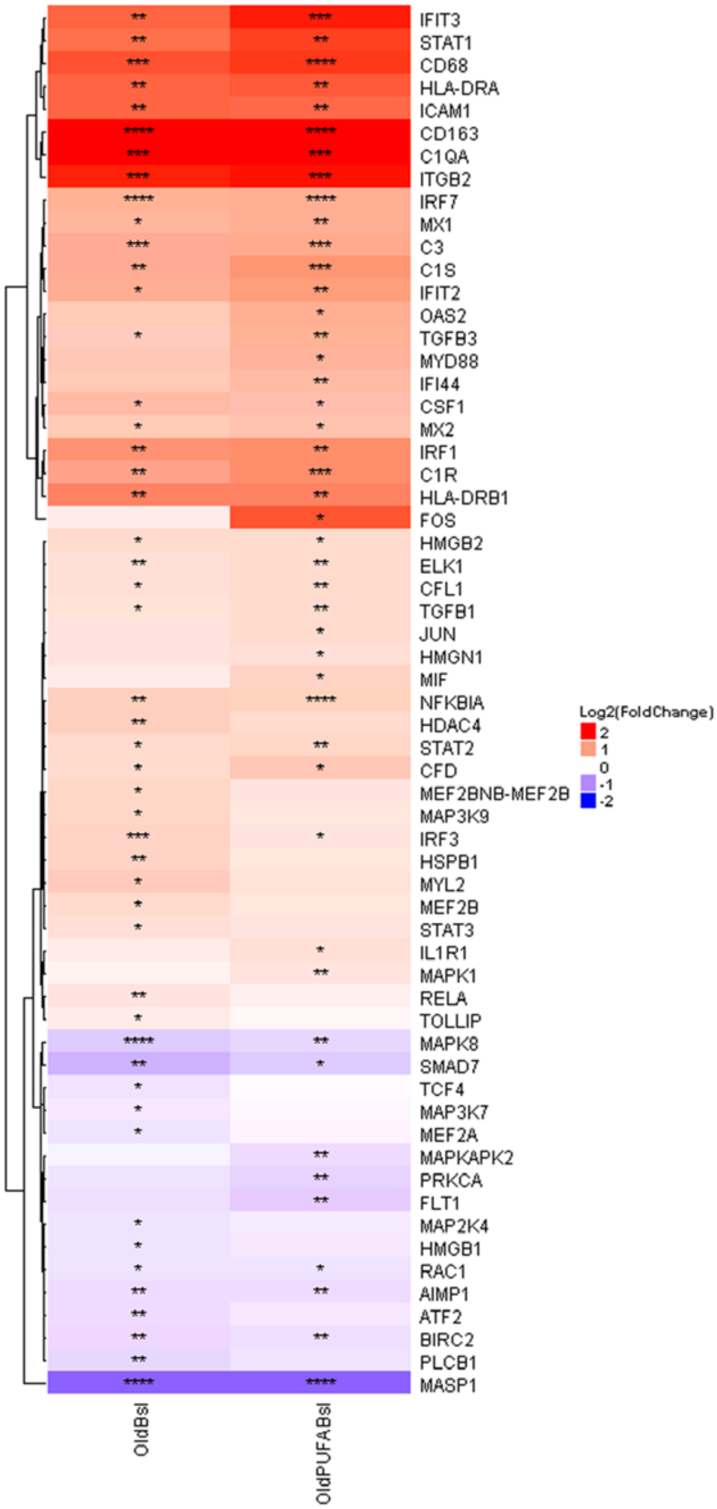

Supplemental Figure 6. The heatmap shows fold changes for genes related to inflammation in old baseline preintervention and old baseline postintervention relative to young baseline. A positive fold change (red) indicates upregulated in old compared to young, and a negative fold change (blue) indicated downregulated in old compared to young. * denotes where the fold change was statistically significant (p≤0.05).

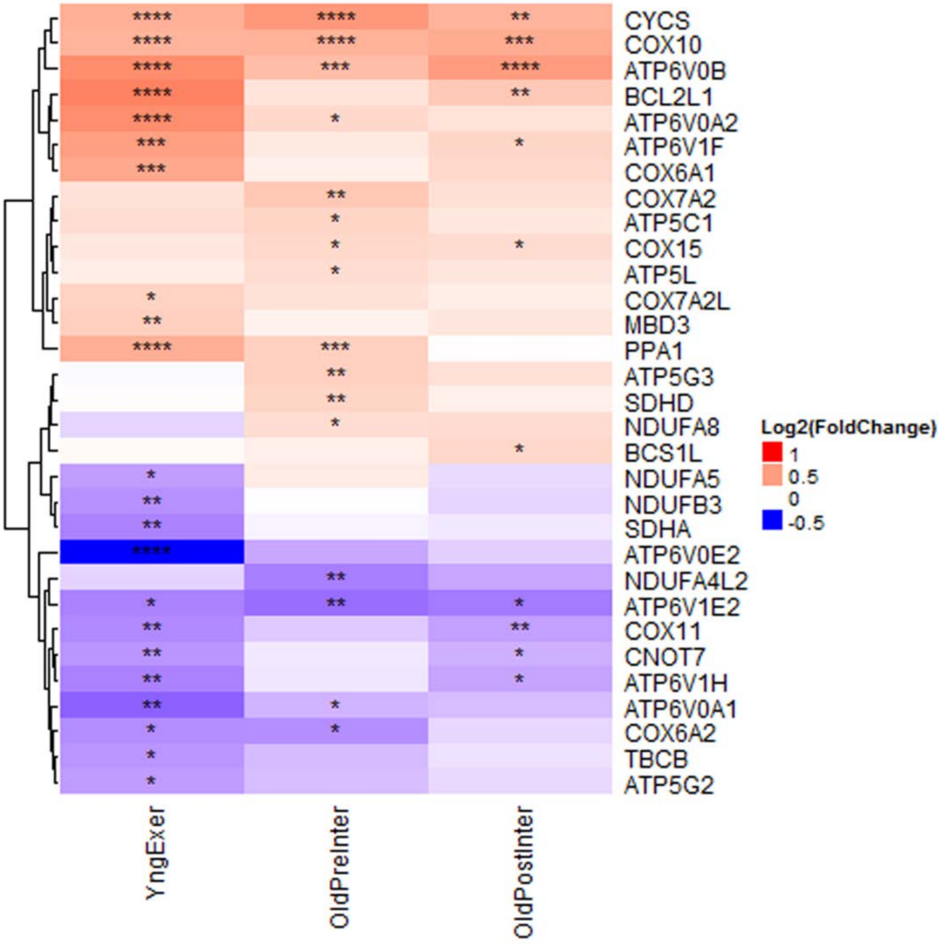

Supplemental Figure 7. The heatmap shows post exercise fold changes for genes related to oxidative phosphorylation in young, old, and old post-supplementation relative to their respective baseline transcript levels. A positive fold change (red) indicates upregulated after exercise, and a negative fold change (blue) indicated downregulated after exercise. * denotes where the fold change was statistically significant from pre-exercise ($p \leq 0.05$).

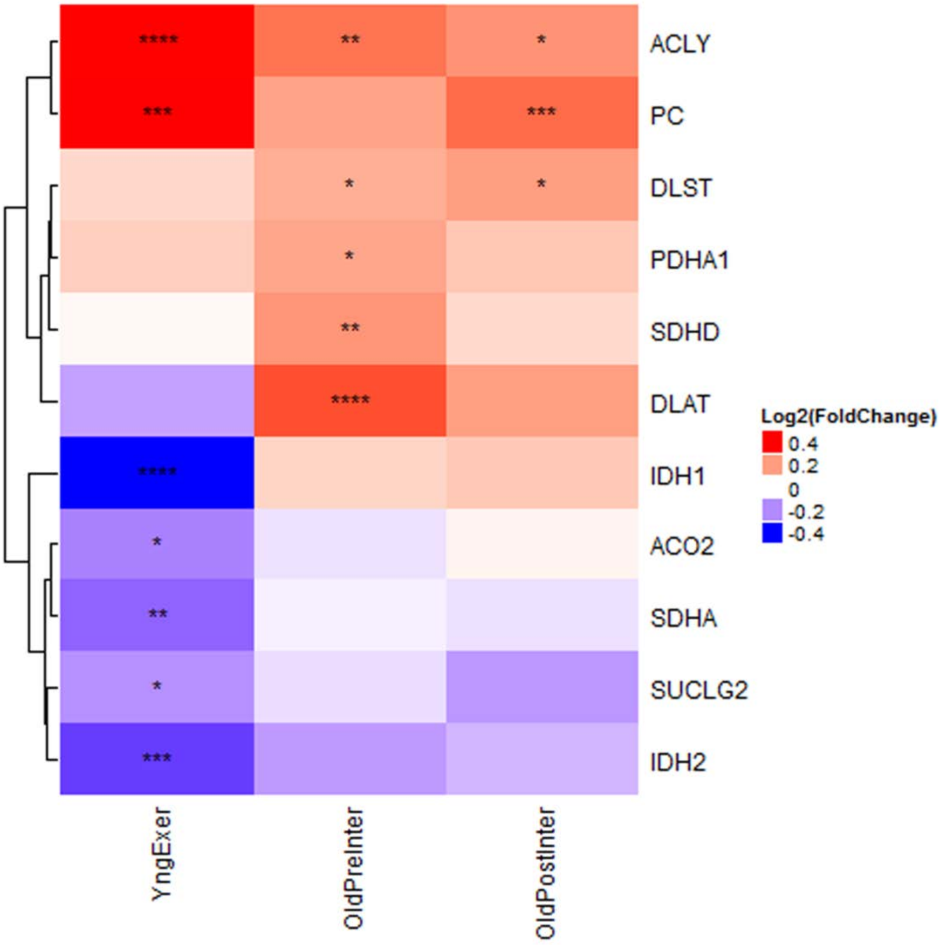

Supplemental Figure 8. The heatmap shows post exercise fold changes for genes related to tricarboxylic acid cycle in young, old, and old post-supplementation relative to their respective baseline transcript levels. A positive fold change (red) indicates upregulated after exercise, and a negative fold change (blue) indicated downregulated after exercise. * denotes where the fold change was statistically significant from pre-exercise (p≤0.05).

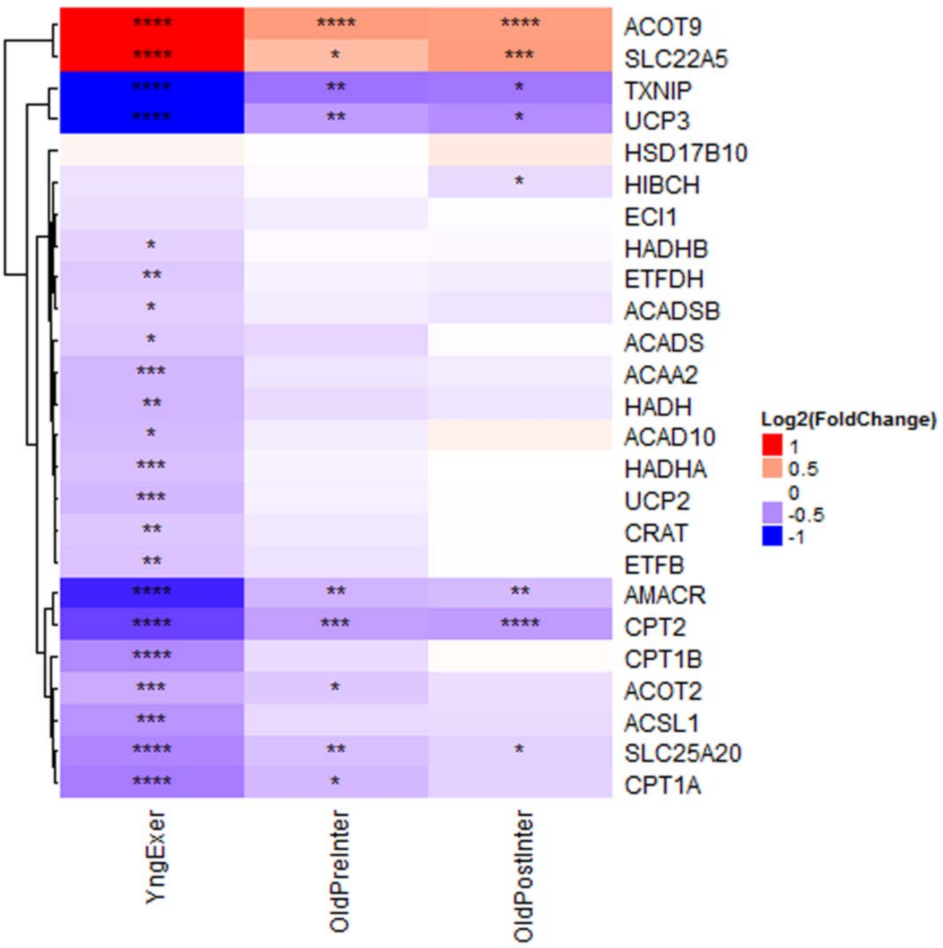

Supplemental Figure 9. The heatmap shows post exercise fold changes for genes related to lipid metabolism in young, old, and old post-supplementation relative to their respective baseline transcript levels. A positive fold change (red) indicates upregulated after exercise, and a negative fold change (blue) indicated downregulated after exercise. * denotes where the fold change was statistically

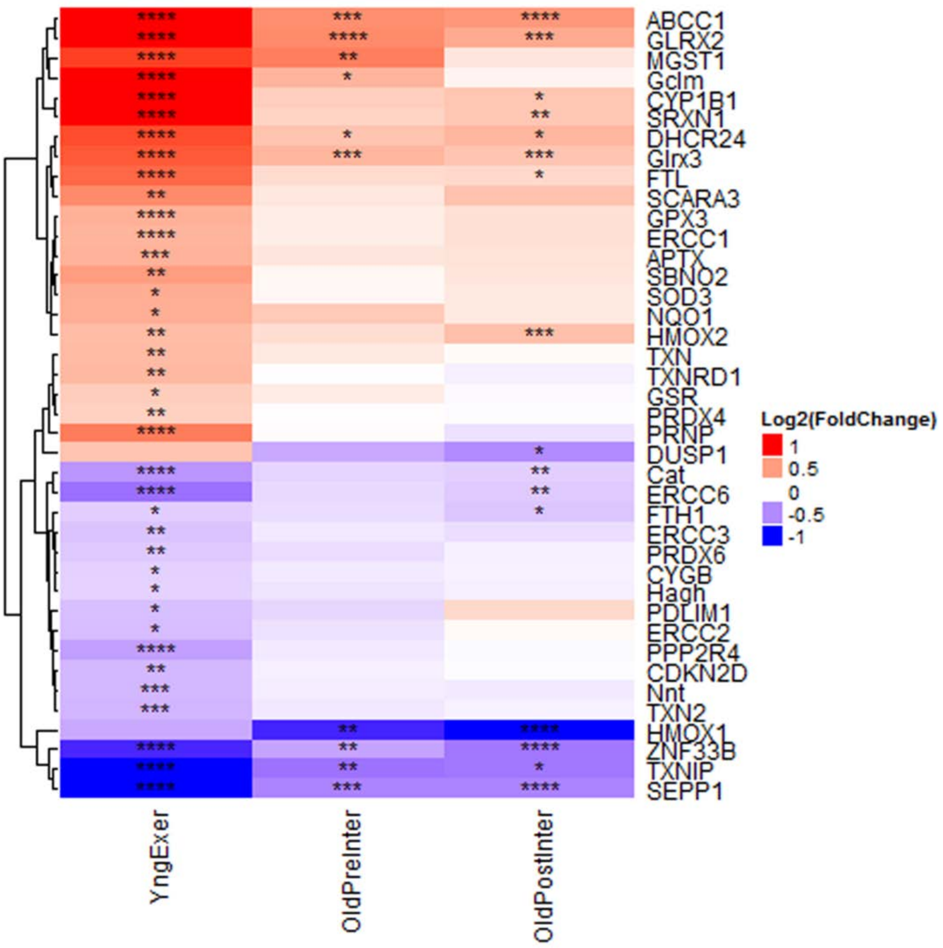

Supplemental Figure 10. The heatmap shows post exercise fold changes for genes related to oxidative stress in young, old, and old post-supplementation relative to their respective baseline transcript levels. A positive fold change (red) indicates upregulated after exercise, and a negative fold change (blue) indicated downregulated after exercise. * denotes where the fold change was statistically significant from pre-exercise (p≤0.05).

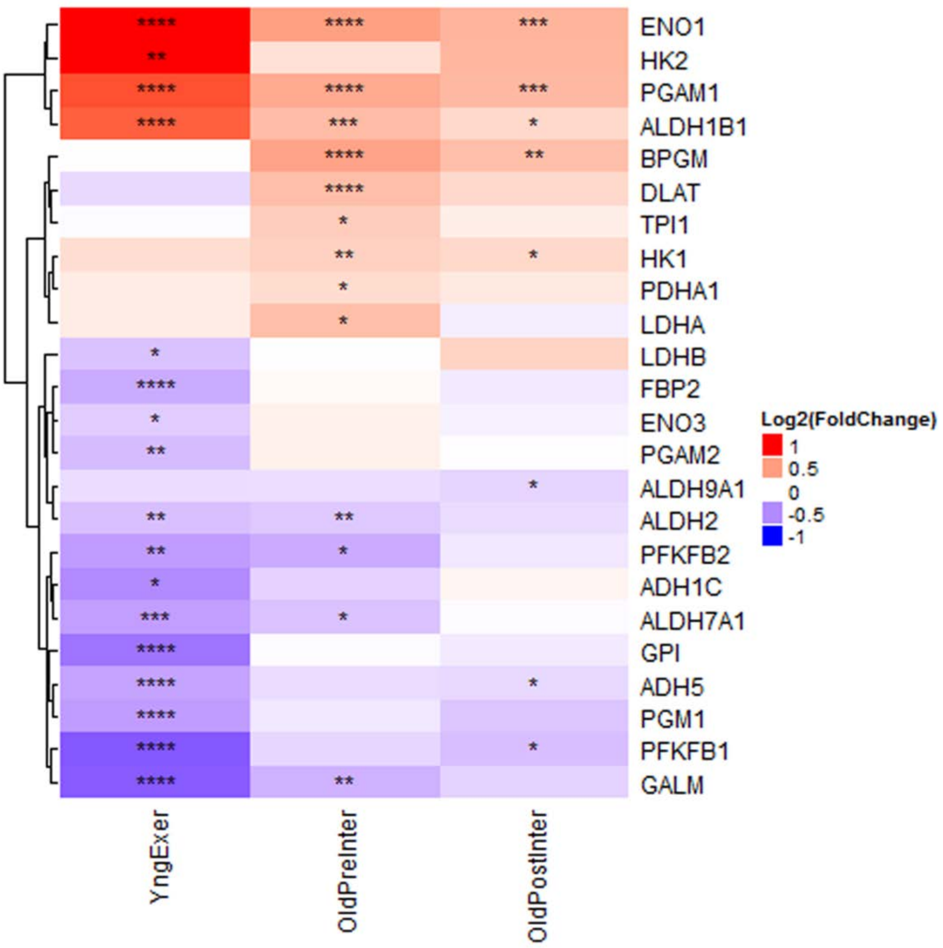

Supplemental Figure 11. The heatmap shows post exercise fold changes for genes related to glycolysis in young, old, and old post-supplementation relative to their respective baseline transcript levels. A positive fold change (red) indicates upregulated after exercise, and a negative fold change (blue) indicated downregulated after exercise. * denotes where the fold change was statistically significant from pre-exercise (p≤0.05).

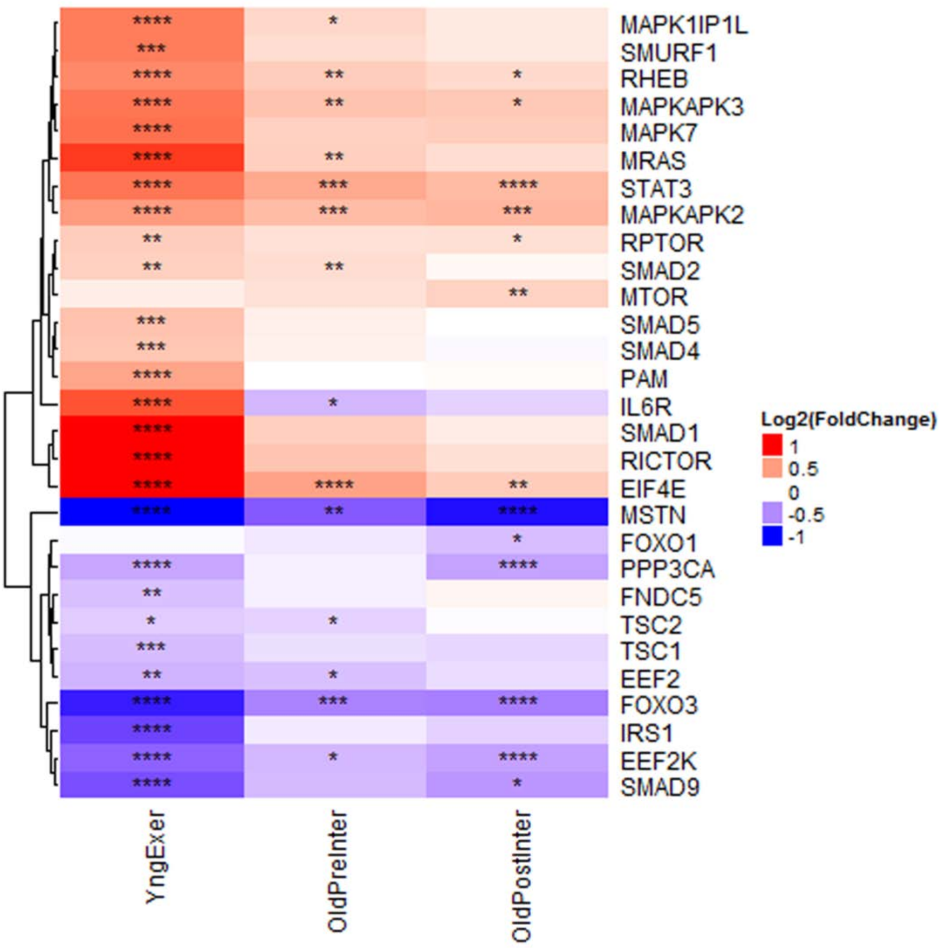

Supplemental Figure 12. The heatmap shows post exercise fold changes for genes related to protein turnover in young, old, and old post-supplementation relative to their respective baseline transcript levels. A positive fold change (red) indicates upregulated after exercise, and a negative fold change (blue) indicated downregulated after exercise. * denotes where the fold change was statistically significant from pre-exercise (p≤0.05).

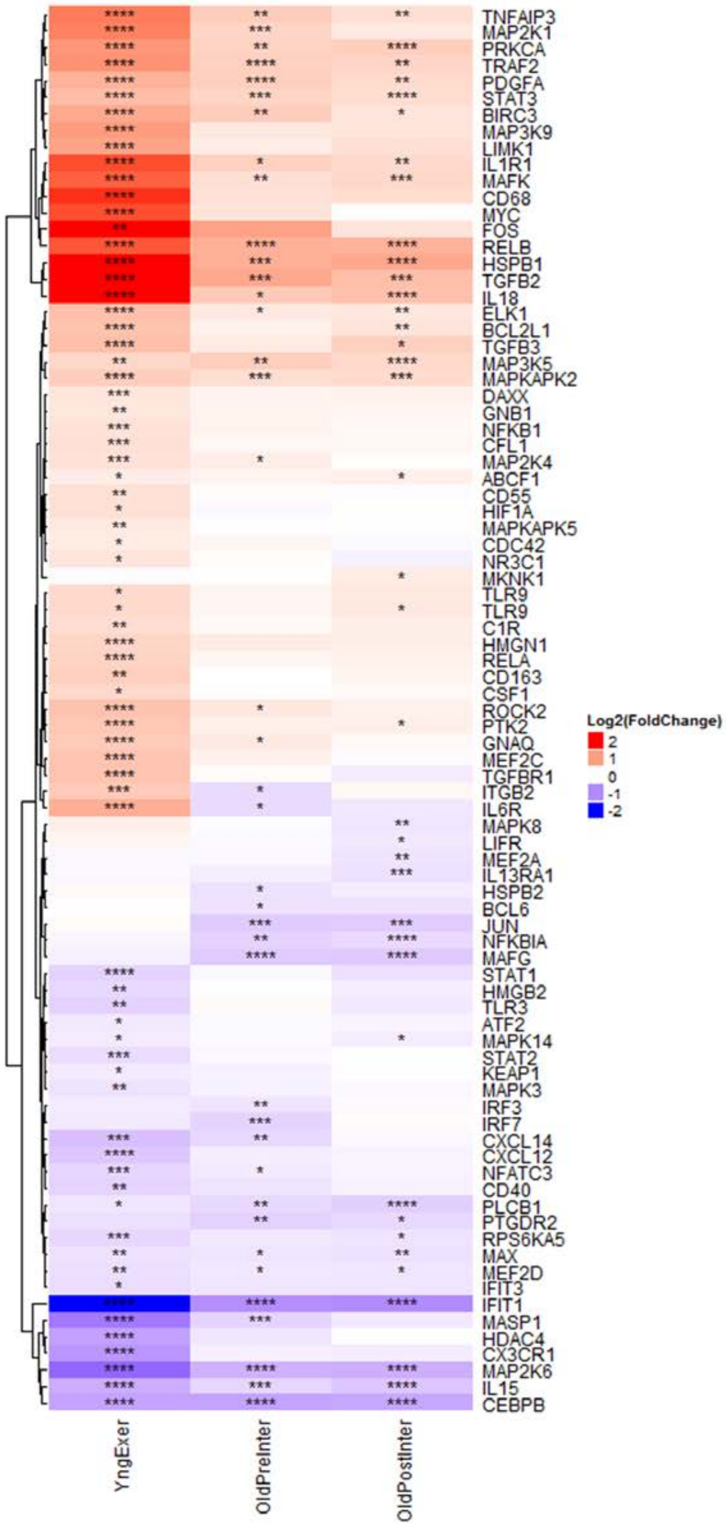

Supplemental Figure 13. The heatmap shows post exercise fold changes for genes related to inflammation in young, old, and old post-supplementation relative to their respective baseline transcript levels. A positive fold change (red) indicates upregulated after exercise, and a negative fold change (blue) indicated downregulated after exercise. * denotes where the fold change was statistically significant from pre-exercise (p≤0.05).

Supplemental Tables

Supplemental Table 1. Gene expression assays.

Gene Symbol	Gene Name	Assay ID	Context Sequence	Probe Dye
• **Gene targets with HPRT1 reference gene (4326321E, VIC probe)**				
SLN	sarcolipin	Hs01888464_s1	ACACGGTCTGCAACC AAACTCTAAT	FAM
FST	follistatin	Hs00246256_m1	TGCCCAGGCTGGGAA CTGCTGGCTC	FAM
CAPN1	calpain 1, (mu/I) large subunit	Hs00559804_m1	AACTACCCAGCCACC TTCTGGGTGA	FAM
TRIM63	tripartite motif containing 63, E3 ubiquitin protein ligase	Hs00822397_m1	GTCGAGTGACCAAGG AGAACAGTCA	FAM
MYOD1	myogenic differentiation 1	Hs02330075_g1	CGACGGCATGATGGA CTACAGCGGC	FAM
• **Gene targets with B2M reference gene (4326319E, VIC probe)**				
B2M	Beta 2 microglobulin			
MSTN	myostatin	Hs00976237_m1	ATGCCTACAGAGTCT GATTTTCTAA	FAM
CAPN2	calpain 2, (m/II) large subunit	Hs00965097_m1	CGGAGCCGAGGAGGT TGAAAGTAAC	FAM
FBXO32	F-box protein 32	Hs01041408_m1	TCCGAGCGGCAGATC CGCAAACGAT	FAM
FOXO3B, FOXO3	forkhead box O3B pseudogene, forkhead box O3	Hs00921424_m1	GGGCAAAGCAGACCC TCAAACTGAC	FAM

Supplemental Table 2. Gene identities of elements in each section of the Venn Diagram in Figure 5.
See Supplemental File 2.
